# Durations of asymptomatic, symptomatic, and care-seeking phases of tuberculosis disease with a Bayesian analysis of prevalence survey and notification data

**DOI:** 10.1186/s12916-021-02128-9

**Published:** 2021-11-10

**Authors:** Chu-Chang Ku, Peter MacPherson, McEwen Khundi, Rebecca H. Nzawa Soko, Helena R. A. Feasey, Marriott Nliwasa, Katherine C. Horton, Elizabeth L. Corbett, Peter J. Dodd

**Affiliations:** 1 Department of Infectious Disease Epidemiology, Imperial College London, London, UK; 2 Malawi-Liverpool-Wellcome Trust Clinical Research Programme, Queen Elizabeth Central Hospital, Blantyre, Malawi; 3 Department of Clinical Sciences, Liverpool School of Tropical Medicine, Liverpool, UK; 4 Department of Clinical Research, London School of Hygiene and Tropical Medicine, London, UK; 5 Helse Nord TB Initiative, University of Malawi College of Medicine, Blantyre, Malawi; 6 Department of Infectious Disease Epidemiology, London School of Hygiene and Tropical Medicine, London, UK; 7 School of Health and Related Research, University of Sheffield, Sheffield, UK

**Keywords:** Tuberculosis, Sub-clinical tuberculosis, Bayesian statistics, Care-seeking, Epidemiology

## Abstract

**Background:**

Ratios of bacteriologically positive tuberculosis (TB) prevalence to notification rates are used to characterise typical durations of TB disease. However, this ignores the clinical spectrum of tuberculosis disease and potentially long infectious periods with minimal or no symptoms prior to care-seeking.

**Methods:**

We developed novel statistical models to estimate progression from initial bacteriological positivity including smear conversion, symptom onset and initial care-seeking. Case-detection ratios, TB incidence, durations, and other parameters were estimated by fitting the model to tuberculosis prevalence survey and notification data (one subnational and 11 national datasets) within a Bayesian framework using Markov chain Monte Carlo methods.

**Results:**

Analysis across 11 national datasets found asymptomatic tuberculosis durations in the range 4–8 months for African countries; three countries in Asia (Cambodia, Lao PDR, and Philippines) showed longer durations of > 1 year. For the six countries with relevant data, care-seeking typically began half-way between symptom onset and notification. For Kenya and Blantyre, Malawi, individual-level data were available. The sex-specific durations of asymptomatic bacteriologically-positive tuberculosis were 9.0 months (95% credible interval [CrI]: 7.2–11.2) for men and 8.1 months (95% CrI: 6.2–10.3) for women in Kenya, and 4.9 months (95% CrI: 2.6–7.9) for men and 3.5 months (95% CrI: 1.3–6.2) for women in Blantyre. Age-stratified analysis of data for Kenya showed no strong age-dependence in durations. For Blantyre, HIV-stratified analysis estimated an asymptomatic duration of 1.3 months (95% CrI: 0.3–3.0) for HIV-positive people, shorter than the 8.5 months (95% CrI: 5.0–12.7) for HIV-negative people. Additionally, case-detection ratios were higher for people living with HIV than HIV-negative people (93% vs 71%).

**Conclusion:**

Asymptomatic TB disease typically lasts around 6 months. We found no evidence of age-dependence, but much shorter durations among people living with HIV, and longer durations in some Asian settings. To eradicate TB transmission, greater gains may be achieved by proactively screening people without symptoms through active case finding interventions

**Supplementary Information:**

The online version contains supplementary material available at 10.1186/s12916-021-02128-9.

## Background

Population surveys of the prevalence of bacteriologically-positive tuberculosis (TB) disease are a key tool for understanding TB epidemiology and burden, and, when repeated over time, for monitoring the impacts of control efforts. (Bacteriologically-positive TB is TB that is diagnosed with a positive result to any bacteriological test: sputum smear, culture or Xpert.) Over the last decade, the World Health Organization (WHO) has encouraged and facilitated a series of nationally-representative TB prevalence surveys in priority countries [1]. Standardisation of methodology and reporting for TB prevalence surveys has been aided by the publication of WHO guidance [2], and detailed methods and results for national TB prevalence surveys are usually published as reports and peer-reviewed articles. TB prevalence surveys are a particularly important source of data for estimating TB incidence in high-burden settings where notification systems are imperfect [1], and while typically powered to achieve a 20% relative precision in the measurement of TB prevalence [2], surveys also contain additional information on subgroups which has, for instance, highlighted the higher burden of TB among men [3].

By comparing prevalence with notifications—usually as a prevalence-to-notification (P:N) ratio—one can estimate a typical timescale for prevalent TB, the inverse of which (the patient diagnostic rate) provides a measurable indicator of the effectiveness of case detection [4]. Comparing P:N ratios between sexes has shown men have poorer access to care in many settings [3]. However, the influence of age and HIV have not been analysed.

Several prevalence surveys also record whether individuals with TB were symptomatic and some record whether individuals with TB had previously sought care for their symptoms [5]. These surveys have found that large proportions of cases—around half—do not report symptoms [6, 7]. Lack of symptoms among those with microbiologically-confirmed pulmonary TB has contributed to an increased understanding of asymptomatic and subclinical TB as being part of a spectrum of TB disease [8], and a potentially important contributor to TB burden and transmission [7].

Many aspects of the natural history of TB disease prior to (or without) treatment remain very uncertain because ethical considerations mean we must rely on historic data from the pre-chemotherapy era. For example, how often and how quickly individuals with smear-negative TB progress to smear-positive disease is unclear. Similarly, while there are data to suggest a typical duration of around three years for untreated TB disease [9], there is only weak and indirect data to quantify the duration of TB among people living with HIV (PLHIV), which is thought to be much shorter [10].

We therefore sought, as our primary focus, to leverage prevalence survey data from a variety of settings to estimate the duration of asymptomatic TB disease and typical delays to care-seeking and notification. Hypothesising an influence of sex, age and HIV infection status on these quantities, our primary objectives also included stratified analyses where data allowed. We used a novel Bayesian framework within which we incorporated uncertainty, disease progression before detection, and trends in incidence. This approach also provided, as by-products, estimates of TB incidence, incidence trends, and case-detection ratios, which we also report as secondary outcomes.

## Methods

### Study populations and data

#### TB prevalence surveys

We analysed data from eleven national and one sub-national setting with a TB prevalence survey conducted after 2010. We excluded TB prevalence surveys that relied solely on symptom screening to decide who to sample for bacteriological testing and restricted to the population aged 15 years or older.

The eleven national settings with recent TB prevalence surveys are Cambodia [11], Ethiopia [12], Kenya [13], Lao PDR [14], Malawi [15], Pakistan [16], Philippines [17], Tanzania [5, 18], Ugand a[19], Vietnam [20], and Zambia [21]. For those prevalence surveys, we extracted the raw counts of eligible subjects and active cases, excluding those already on TB treatment if possible. Seven surveys included healthcare behaviour data enabling us to identify whether symptomatic cases had started seeking care.

Summaries of prevalence survey data are shown in the upper part of Table 1. The proportion of prevalent cases that were asymptomatic ranged from 30% in Malawi, to 70% in Cambodia, and the proportion of prevalent cases that were sputum smear-positive varied from 23% in Vietnam to 84% in Tanzania. Definitions used for symptoms, care-seeking, and raw counts extracted from the prevalence are summarised in Additional file 2.

**Table 1:** Epidemiological estimates. World Health Organization (WHO) case detection ratio (CDR), our CDR, proportions of cases dying and self-curing before treatment, rates of decline by setting

	Cambodia	Ethiopia	Kenya	Lao PDR	Malawi	Pakistan	Philippines	Uganda	Tanzania	Vietnam	Zambia
Population aged 15 and above, 2019, million	11.4	66.9	32	4.9	10.5	140.6	75.2	23.7	32.6	74.1	9.9
**Prevalence survey**											
Survey year	2011	2010–2011	2016	2010–2011	2013	2010–2011	2016	2014–2015	2012	2017–2018	2013–2014
Prevalence in the survey year, per 100,000^a^	839 (748–933)	236 (193–281)	478 (425–532)	604 (528–683)	418 (348–491)	325 (291–360)	998 (908–1090)	395 (335–456)	305 (258–355)	358 (311–406)	575 (508–644)
**WHO data/estimates**											
Case notification, 2019, per 100,000	247 (244–250)	150 (149–150)	238 (236–240)	138 (135–142)	146 (144–148)	201 (200–202)	488 (486–489)	238 (236–240)	212 (210–213)	136 (135–137)	340 (336–343)
Case-detection ratio	57 (40.0–88)	69 (51.0–98)	63 (43.0–100)	57 (40.0–89)	56 (35.0–110)	64 (48.0–90)	63 (40.0–110)	65 (43.0–110)	53 (30.0–110)	57 (40.0–90)	58 (41.0–90)
Incidence rate in 2019, per 100,000	387 (220–546)	208 (133–283)	385 (194–572)	206 (120–288)	218 (85–361)	351 (225–478)	705 (310–1102)	304 (135–473)	356 (98–617)	219 (127–312)	534 (302–766)
**Model estimates: All TB regardless of symptomatic or not**											
Duration by P:N ratio, month^b^	28.9 (25.8–32.3)	10.2 (8.4–12.3)	25.1 (22.5–28.0)	109.4 (95.1–125.6)	29.7 (25.1–34.9)	17.8 (15.9–19.9)	33.3 (30.5–36.4)	24.5 (21.0–28.5)	20.8 (17.7–24.2)	33.0 (28.6–37.3)	16.0 (14.2–18.0)
Time bacteriologically positive, month	22.0 (18.9–25.8)	9.0 (7.5–10.8)	18.3 (16.1–21.0)	36.1 (29.9–45.3)	21.8 (18.4–25.9)	13.1 (11.5–14.8)	22.3 (19.6–25.6)	19.1 (16.4–22.2)	16.2 (14.0–18.8)	20.7 (17.8–24.5)	13.4 (11.8–15.2)
Case-detection ratio^c^	67% (56–79%)	87% (80–94%)	69% (60–79%)	24% (19–31%)	63% (53–74%)	76% (68–84%)	53% (46–62%)	63% (53–72%)	68% (59–77%)	59% (50–70%)	79% (71–88%)
Incidence rate in 2019, per 100,000	342 (285–403)	168 (156–183)	360 (312–414)	595 (449–743)	222 (187–265)	287 (259–319)	914 (782–1,055)	354 (305–412)	307 (271–352)	230 (194–272)	426 (382–474)
**Model estimates: All symptomatic TB**											
Duration by P:N ratio, month	8.7 (7.1–10.5)	5.3 (4.0–6.7)	11.8 ( 9.9–13.8)	53.4 (43.6–64.1)	20.6 (16.7–24.9)	10.9 (9.4–12.4)	10.9 (9.2–12.7)	11.9 (9.4–14.6)	13.3 (10.7–16.0)	21.9 (18.6–25.6)	9.7 (8.2–11.3)
Time without care-seeking, month	1.2 (0.6–1.9)	N.A.	5.6 (4.6–6.8)	N.A.	6.6 (4.8–8.6)	N.A.	4.2 (3.3–5.2)	4.2 (3.0–5.6)	6.3 (4.9–7.8)	N.A.	4.1 (3.3–5.1)
Time bacteriologically positive, month	8.0 (6.7– 9.6)	4.8 (3.7–6.0)	9.5 (8.2–11.1)	21.6 (18.0–26.8)	15.8 (13.2–19.0)	8.2 (7.2–9.4)	9.2 (7.9–10.6)	10.4 (8.5–12.5)	10.9 (9.1–13.0)	14.2 (12.1–16.8)	8.5 (7.3– 9.8)
Case-detection ratio	83% (77–89%)	92% (87–96%)	80% (73–86%)	36% (29–44%)	69% (59–79%)	82% (76–88%)	75% (69–81%)	75% (67–82%)	75% (67–82%)	66% (57–75%)	85% (78–91%)
Incidence rate in 2019, per 100,000	270 (252–292)	160 (154–169)	309 (286–338)	396 (315–482)	203 (177–235)	265 (247–285)	643 (593–700)	296 (269–329)	277 (252–309)	206 (181–235)	397 (369–429)
**Other estimates/metrics**											
Annual decline rate, % per year	4.7% (4.4–5.0%)	7.3% (7.1–7.5%)	1.5% (1.3–1.6%)	− 8.0% (− 8.5% to − 7.5%)	2.9% (2.6–3.2%)	1.6% (1.5–1.7%)	− 8.3% (− 8.4% to − 8.2%)	− 2.4% (− 2.7% to − 2.2%)	− 1.3% (− 1.5% to − 1.1%)	1.0% (0.7–1.4%)	4.9% (4.7–5.1%)
Asymptomatic phase over total duration, %	64% (58–69%)	47% (39–56%)	48% (42–53%)	40% (35–45%)	27% (21–35%)	37% (32–42%)	59% (53–65%)	45% (38–53%)	32% (26–40%)	31% (26–37%)	37% (31–43%)
Symptomatic TB without care-seeking, %	15% (9–23%)	N.A.	59% (51–67%)	N.A.	42% (33–51%)	N.A.	46% (38–54%)	41% (31–50%)	57% (48–67%)	N.A.	48% (40–56%)
Self-cure before case-detection	32% (20–42%)	14% (8–21%)	27% (16–36%)	50% (35–61%)	30% (19–41%)	20% (12–28%)	32% (20–42%)	27% (17–37%)	23% (14–33%)	30% (19–41%)	20% (12–28%)
Deaths before case-detection	7% (6–9%)	3% (2–4%)	6% (5–7%)	19% (15–26%)	10% (8–12%)	6% (5–7%)	6% (5–7%)	8% (6–10%)	8% (6–9%)	12% (10–14%)	5% (4–6%)

^a^Values are crude rates or proportions with 95% Binomial confidence intervals, while the model estimates are mean estimators with 95% credible intervals

^b^P:N ratios use prevalence from the model estimates for 2019 and notified cases from WHO 2019 TB notification data

^c^Case-detection ratio is defined as the ratio of notified cases and the estimated incident cases in percentage

The sub-national TB prevalence survey from Blantyre, Malawi, and the national survey from Kenya included individual data, allowing analysis by smear status, age, and HIV status [13]. In Blantyre, Malawi, the prevalence survey was conducted as part of a community cluster randomised trial across approximately 750,000 adults in 72 densely populated neighbourhoods of the city. For Blantyre, we aligned symptom definition with that of Kenya by considering cough of ≥ 2 weeks, considering cough of any duration in a sensitivity analysis.

In 2019, a TB prevalence survey was carried out in Blantyre as part of a cluster-randomised trial of community-based TB screening interventions (ISRCTN11400592). Blantyre City was demarcated into 72 neighbourhood clusters, each with approximately 4000 adults aged 15 years or older. All households were enumerated and 115 households per cluster were chosen at random for participation into the prevalence survey, with the aim of recruiting 215 adults (≥ 15 years old) per neighbourhood. Adults from the randomly selected households were visited and invited to visit a study tent where TB symptom screening and digital chest radiograph (interpreted by radiographers and computer-assisted diagnostic software [Qure.ai version 2.0]) were done. Participants who had an abnormal chest X-ray or reported cough of any duration were asked to provide two spot sputum samples for Xpert, smear microscopy, and MGIT culture, and participants with positive results were linked to treatment.

#### Definitions

Unless otherwise stated, we used the definition of TB symptoms and health-care seeking adopted by each TB prevalence survey (see Additional file 2). Asymptomatic TB was taken to be bacteriologically-positive pulmonary TB in those reporting no TB symptoms, and we assumed no health-care seeking for TB during this phase. Participants with prevalent TB who were already taking TB treatment were excluded. We assumed TB treatment initiation, TB confirmation, and case notification are identical events for the modelling.

#### TB notification data

We used TB notification data and treatment outcome data from the WHO TB database for the countries with national prevalence surveys [5, 11–21]. We included new and relapse TB regardless of smear status since 2013. We applied the proportions of HIV among TB notifications by sex, age group, and smear status to disaggregate the WHO notification data. Where notification data exhibited level shifts suggestive of changes in reporting, only consistent, sequential data were used (see graphs in Additional file 1).

In order to take advantage of the finer-grained prevalence data for Kenya and Blantyre, Malawi, we sought correspondingly stratified notification data. For Kenya, we obtained data simultaneously stratified by HIV and age group from the National Tuberculosis Programme. For Blantyre, we obtained individual-level notification data from an enhanced surveillance system. We matched the age-groups of the Kenya data to the finest scale of WHO case notification data; for Blantyre, Malawi, with lower case counts, we used two age groups (15 to 49, and 50+ years).

#### Demographic data

In order to model mortality and project national numbers of prevalent cases to relate to notifications, we used age- and sex-specific background mortality rates and population sizes from 2019 World Population Prospect (WPP) data, using the mid-year population estimates [22]. For Blantyre, we rescaled the Malawi demographic data to local 2008 and 2018 population censuses [23, 24].

Additional file 2 details the extracted data and information from the prevalence surveys, and the demographic and notification data to replicate the analysis are available at Addition file 3, 4 and 5.

### Statistical methods

#### Estimation of TB progression and care-seeking behaviour

**Fig. 1:**
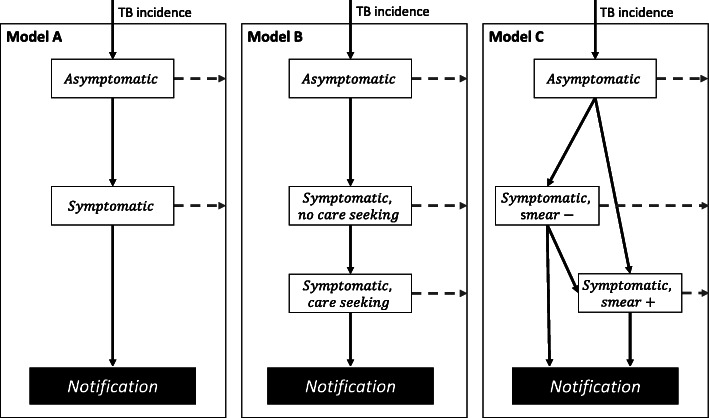
Diagrams of model structure. White boxes are states representing all bacteriologically-positive TB accessible to prevalence surveys, with structure matched to available data. Dashed arrows represent transitions out of active disease, either through self-cure or death. Models are additionally stratified by sex, and for Kenya and Blantyre, Malawi by age and HIV in addition

We developed three state transition models of TB case-detection to match available data, and fitted them to estimate parameters driving TB progression and care-seeking behaviour. Figure 1 shows that all models contain an asymptomatic phase and progress to the symptomatic phase. Model A presents a basic transition between asymptomatic/symptomatic phases; Model B divides the symptomatic phase by presence of care-seeking intentions, and Model C details the conversion between smear-negative and smear-positive and care-seeking by smear status. Model B is only applicable to the prevalence surveys that reported care-seeking behaviours. We did not combine Model B and C because the symptoms, smear status, and care-seeking behaviours prevalence surveys were not cross-tabulated in the published reports. We constructed a likelihood depending on these states for fitting to TB prevalence and case-notification data. For Kenya and Blantyre data with Model C, we incorporated proportional hazard models relating the rate of developing symptoms and the care-seeking rates by smear status to covariates of age, sex, and HIV status. The choice of model type used was determined by which data were available in the prevalence survey. These model structures follow the usual structures used in conventional ordinary differential equation TB models to describe the progression and detection or prevalent TB, e.g. the smear status-stratified model introduced by Dye et al. [25], but also including symptom and care-seeking progression similarly to Dowdy et al. [26]

We calculated a single weighted-mean background mortality rate from WPP data in each country, using WHO TB notifications as weights. For the analyses with HIV stratification, we added HIV-related deaths for PLHIV from the UNAIDS database: 0.016 per year (21,000/1,300,000) for Kenya in 2016 and 0.011 per year (11,000/1,000,000) for Blantyre (based on Malawi data). In asymptomatic TB, we assumed no TB-related deaths, but did allow self-cure and deaths due to other causes. Smear status-specific TB death rates were applied to symptomatic TB states based on Tiemersma et al. [9]. For Model A and Model B, regardless of smear status, we applied the untreated TB deaths weighted by smear status in the notification data. We conducted sensitivity analyses for assumed excess mortality rates of asymptomatic/symptomatic TB. We also considered two extreme assumptions for HIV-TB excess mortality: the same TB mortality rate as HIV-negative people, and an excess mortality rate of 5% per month as considered in Vynnycky et al. [27] for gold miners in South Africa. Finally, we considered the impact on estimates of fitting to data from mis-specified models allowing symptom reversion.

We assumed each state declined exponentially with a constant shared rate. The statistical models were constructed within a Bayesian framework; Additional file 1 details the mathematical formulation and priors. We fitted all models by Markov chain Monte Carlo (MCMC) using R with RStan. Inferences were based on 3000 samples from three chains. For each chain, we set at least 4000 burn-in steps and increased thinning to ensure the effective sample sizes are larger than 10% of sample sizes. Processed data, all source code, and detailed diagnostics for this analysis are available on Github as [https://github.com/TimeWz667/AsymTB].

#### Metrics calculated

For each setting, we used posterior samples to calculate: the TB incidence; the mean durations of asymptomatic disease, disease without case-seeking initiation (where applicable), and prevalent untreated disease; the case detection ratio (CDR; defined as the ratio of estimated incidence rates and observed notification rates) as the ratio of incidence and notification rates; and the proportion of TB cases reaching each stage of care (assuming unidirectional progression through states). Aggregated quantities were computed weighted by model-estimated incidence. We also output a joint posterior for the proportion of symptomatic cases initially smear-positive and the subsequent smear-conversion rate. We report means and 95% credible intervals (CrIs).

## Results

MCMC runs all converged with Gelman-Rubin R^2 statistics < 1.05. Supplementary data on convergence and parameter posteriors are presented in Additional file 1.

### Duration of asymptomatic disease, time without care-seeking initiation and time to case-detection

**Fig. 2:**
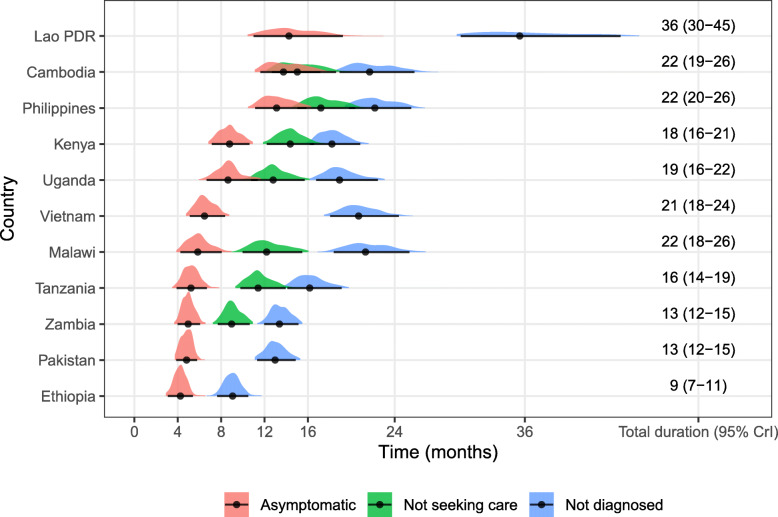
Total time in months spent in each state during bacteriologically-positive TB disease. ‘Not diagnosed’ includes all states (white boxes) in Fig. 1. Median and 95% quantiles are shown as points and error bars, respectively. Posterior distributions are shown by coloured kernel density estimates

The national estimates for duration of asymptomatic disease typically ranged around 3–8 months. However, three countries in Asia (Lao PDR, Cambodia, Philippines) showed longer durations of over one year (see Fig. 2). These countries also had long total durations: 22 months for Cambodia and Philippines, and three years for Lao PDR. Only one country (Ethiopia) had a total duration lower than 12 months. In the seven countries where we could estimate the delay to care-seeking initiation, apart from the Philippines (16 months), delay varied between 1.2 (0.7–1.9) months for Cambodia and 6.6 (95% CrI: 4.8–8.6) months for Malawi. The asymptomatic phase represented between 27% and 63% of time as a prevalent case, and care-seeking occurred between 15% and 59% of the way between the first symptom developed and notification (or death or self-cure).

### Development of smear-positivity

**Fig. 3:**
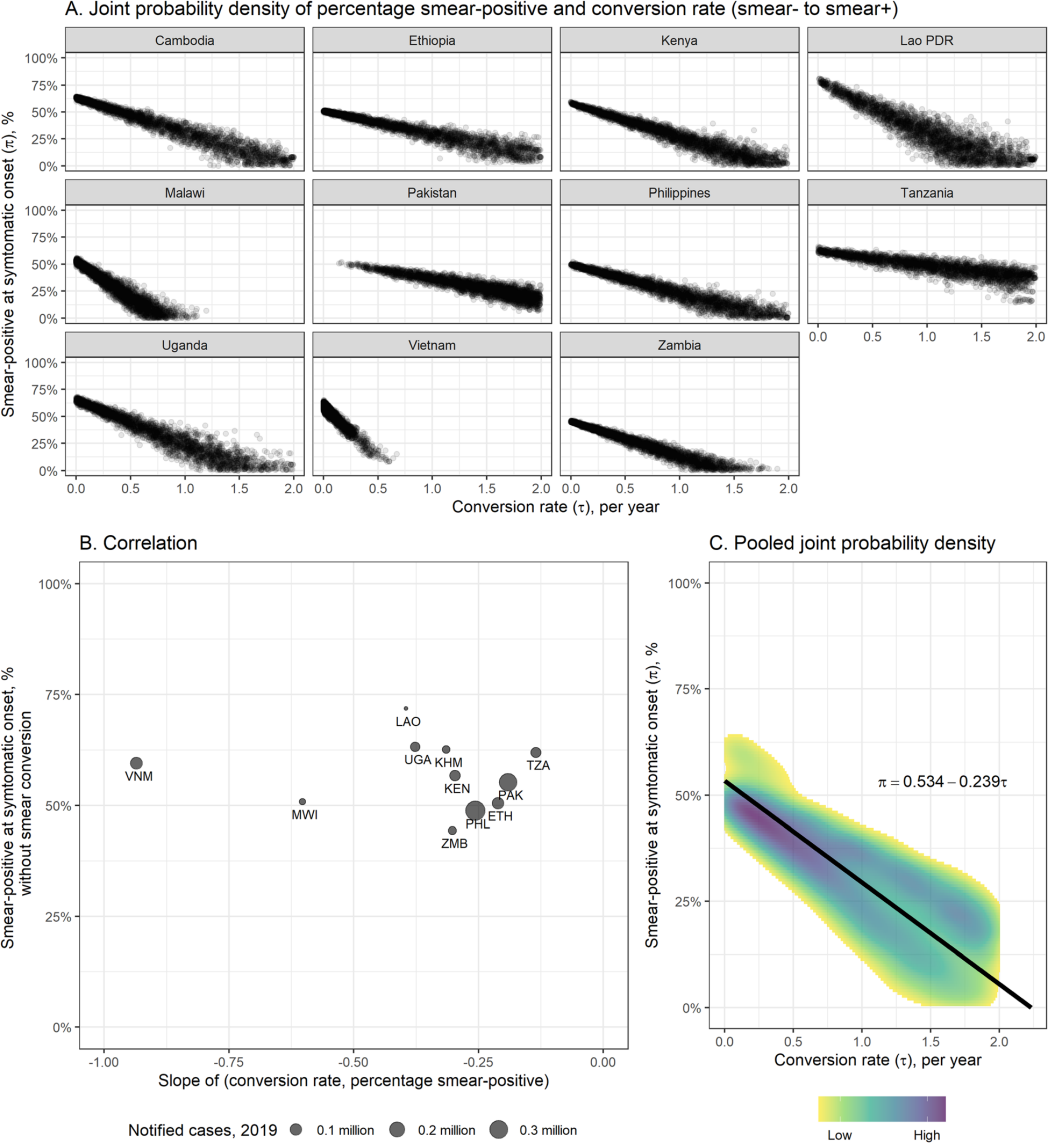
Smear-conversion rate and initial proportion smear-positive in symptomatic bacteriologically-positive TB disease. **A** joint posterior probability densities of initial proportion smear-positive (*y*-axis) and hazard of converting from smear-negative to smear-positive (*x*-axis) in symptomatic bacteriologically-positive TB by country, based on Model 3. **B** the correlation of percentage smear-positive at symptom onset and smear-type conversion rate. X-axis indicates the slopes of **A** estimated by linear regression (KHM = Cambodia, ETH = Ethiopia, KEN = Kenya, LAO = Lao People’s Democratic Republic, MWI = Malawi, PAK = Pakistan, PHL = Philippines, TZA = United Republic of Tanzania, UGA = Uganda, VNM = Viet Nam, ZMB = Zambia) (**C**) joint probability density of initial proportion smear-positive (*y*-axis) and hazard of converting from smear-negative to smear-positive (*x*-axis) pooled by weights of notified cases in 2019 (excluding Malawi and Vietnam as outliers)

Figure 3 shows the joint posterior probability densities of smear conversion rates and the proportion of symptomatic cases that were smear-positive at symptom onset. Estimates of smear conversion rates were linearly related to the initial proportion of symptomatic cases that were smear-positive. Pooling weighted by TB notifications in 2019 (excluding Malawi and Vietnam as outliers) yielded a gradient of 0.239 years, and a pooled intercept of 53.4% smear-positive (for zero smear conversion rate).

### Estimates of epidemiological indices considering asymptomatic phase of TB

Table 1 shows the empirical estimates of total duration and total asymptomatic duration based on P:N ratios. Empirical estimates of duration systematically overestimate the durations (see also direct comparison of additional results in Additional file 1), because they implicitly assume all episodes of TB disease end in notification. Modelling self-cure and death leads to shorter estimates of duration and differences in estimated CDRs from WHO estimates. The proportions of incident TB who die or self-cure before being detected are shown in Table 1. Estimates of TB incidence are also distinct but comparable to WHO incidence estimates. We estimated rates of TB incidence change from a decline of 7.3% (95% CrI: 7.1–7.5%) per year in Ethiopia up to an increase of 8.3% (95% CrI: 8.2–8.4%) per year in the Philippines.

### Care-seeking cascade estimates

**Fig. 4:**
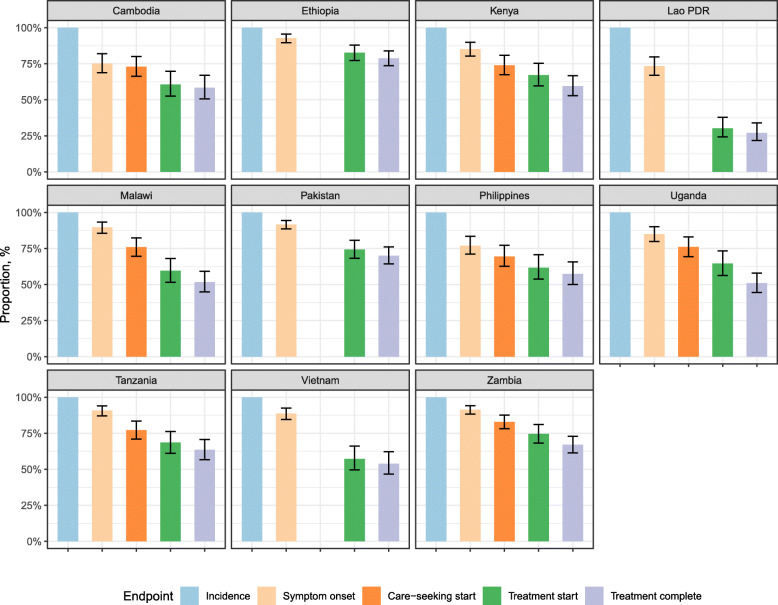
Healthcare cascade. The values are the fraction of the incident cohort reaching each stage. The second bars may be missing if the TB prevalence survey did not report results on care-seeking behaviour

Figure 4 shows care cascade estimates for the year of national prevalence surveys, showing the proportion of incident TB cases that develop symptoms, begin seeking care, initiate treatment, and finally successfully complete treatment, assuming unidirectional progression. There were notable differences between countries, with the proportion initiating TB treatment ranging from 30% (95% CrI: 24–38%) in Lao PDR to 83% (95% CrI: 72–88%) in Ethiopia. Versions of these figures with cohort timings are shown in Additional file 1.

### Sex, age, and HIV

For Kenya and Blantyre, Malawi, we were able to examine results stratified by sex, age, and HIV-status (see Table 2). For Kenya we found total durations of 18.8 months (95% CrI: 16.1–21.8) for men and 15.4 months (95% CrI: 13.0–18.1) for women, and for Blantyre 9.9 months (95% CrI: 6.7–13.8) for men and 6.6 months (95% CrI: 3.5–10.2) for women, in line with previous work suggesting longer durations for men [3].

**Table 2:** Covariate analysis for symptom development rate for Kenya and Blantyre. Asymptomatic, symptomatic and total TB duration by sex, age and HIV status for Kenya and Blantyre, Malawi (RR = risk ratio)

			Asymptomatic duration, month	Symptom onset rate after TB activation, per year			Symptomatc duration, month	Total duration, month
				Crude rate	Crude RR	Adjusted RR		
Kenya	Overall		8.3 (6.8–10.1)	1.25 (1.05–1.50)			8.4 (7.1– 9.7)	16.7 (14.5–19.1)
	Sex	Female	8.1 (6.2–10.3)	1.30 (1.01–1.69)	Reference	Reference	7.2 (5.8–8.8)	15.4 (13.0–18.1)
		Male	9.0 (7.2–11.2)	1.14 (0.93–1.40)	0.89 (0.63–1.20)	0.68 (0.50–0.89)	9.7 (8.2–11.5)	18.8 (16.1–21.8)
	Age	(15,25)	9.5 (6.6–13.0)	1.11 (0.76–1.60)	reference	reference	10.4 (7.9–13.2)	20.0 (16.1–24.4)
		(25,35)	8.6 (6.3–11.4)	1.23 (0.90–1.66)	1.13 (0.75–1.67)	1.20 (0.77–1.69)	9.4 (7.5–11.5)	18.0 (15.0–21.6)
		(35,45)	7.1 (5.0– 9.7)	1.55 (1.08–2.22)	1.43 (0.87–2.17)	1.72 (1.10–2.54)	7.7 (5.9– 9.7)	14.7 (11.9–18.1)
		(45,55)	7.4 (4.8–10.7)	1.49 (0.94–2.31)	1.39 (0.80–2.43)	1.82 (0.95–3.17)	8.3 (6.0–11.1)	15.7 (12.4–19.7)
		(55,65)	9.6 (6.1–13.7)	1.10 (0.70–1.74)	1.02 (0.55–1.72)	1.23 (0.68–2.06)	5.2 (3.1–8.3)	14.8 (10.9–19.4)
		(65,Inf)	7.1 (4.6–10.2)	1.52 (0.97–2.35)	1.43 (0.80–2.55)	1.42 (0.89–2.34)	5.3 (3.4–7.7)	12.4 (9.4–16.0)
	HIV	Non-HIV	9.8 (7.9–12.2)	1.03 (0.85–1.25)	Reference	Reference	9.9 (8.5–11.7)	19.7 (17.1–23.0)
		PLHIV	3.8 (2.4–5.6)	3.09 (1.96–4.80)	2.88 (1.82–4.54)	2.45 (1.45–3.68)	4.0 (2.5–5.7)	7.8 (5.8–10.2)
Blantyre	Overall		3.8 (2.3–5.8)	3.10 (1.86–4.98)			3.7 (2.3–5.4)	7.5 (5.4–10.1)
	Sex	Female	3.5 (1.3–6.2)	3.90 (1.73–8.83)	reference	reference	3.1 (1.3–5.5)	6.6 (3.5–10.2)
		Male	4.9 (2.6–7.9)	2.46 (1.34–4.48)	0.82 (0.29–1.82)	0.81 (0.30–1.71)	5.0 (2.9–7.7)	9.9 (6.7–13.8)
	Age	(15,50)	4.0 (2.3–6.2)	3.00 (1.74–5.03)	reference	reference	3.7 (2.1–5.6)	7.7 (5.3–10.3)
		(50,Inf)	3.5 (1.1–7.1)	4.17 (1.46–10.84)	1.53 (0.45–3.94)	1.41 (0.47–4.16)	4.6 (1.9–8.1)	8.1 (4.3–12.5)
	HIV	Non-HIV	8.5 (5.0–12.7)	1.29 (0.76–2.16)	reference	reference	7.1 (4.3–10.8)	15.6 (11.3–20.8)
		PLHIV	1.3 (0.3–3.0)	12.79 (3.77–41.16)	5.33 (1.90–11.92)	4.81 (1.65–12.82)	2.4 (1.0–4.1)	3.7 (1.9–6.0)

*RR* risk ratio, *PLHIV* people living with HIV

Adjusted results for Kenya suggested longer durations asymptomatic in men than women; however, there were no clear differences in unadjusted results and results from Blantyre (see risk ratios in Table 2). There were also no clear patterns with respect to age. However, HIV status had a strong effect, with asymptomatic and symptomatic durations being shorter in PLHIV than those that were HIV negative. The total duration in PLHIV was 3.7 months (95% CrI: 1.9–6.0) for Blantyre and 7.8 months (95% CrI: 5.8–10.2) for Kenya. We found that CDRs were consistently higher in PLHIV than in those without HIV infection, even with higher deaths rates for PLHIV with TB assumed (Table 3).

**Table 3:** Epidemiological estimates by HIV status, Kenya and Blantyre. TB incidence, case detection ratio, and smear-stratified symptom duration by HIV status for Kenya and Blantyre, Malawi

	Kenya			Blantyre		
HIV status	Non-HIV	PLHIV		Non-HIV	PLHIV	
Background mortality	μ	μ + HIV-related mortality		μ	μ + HIV-related mortality	
excess mortality for symptomatic TB	Smear−: 50%/10 years Smear+: 70%/10 years		5% per month	Smear−: 50%/10 years Smear+: 70%/10 years		5% per month
**All TB regardless of symptomatic or not**						
Time asymptomatic, month	10.35 (8.41–12.84)	3.81 (2.34–5.51)	3.22 (1.99–4.75)	8.79 (5.14–13.18)	1.32 (0.31–3.04)	1.15 (0.25–2.64)
Time bacteriologically positive, month	20.90 (18.01–24.46)	7.79 (5.78– 9.99)	6.49 (4.94–8.11)	16.27 (11.59–21.53)	3.76 (1.81–6.07)	3.26 (1.76–5.03)
Case-detection ratio	69% (59–81%)	84% (77–91%)	71% (63–80%)	76% (64–88%)	94% (88–98%)	84% (74–93%)
Incidence rate in 2019, per 100,000	263 (221–306)	2025 (1873–2205)	2403 (2142–2715)	158 (132–192)	1742 (1618–1879)	1949 (1745–2217)
**All symptomatic TB**						
Time bacteriologically positive, month	10.56 (8.86–12.48)	3.99 (2.52–5.56)	3.27 (2.16–4.40)	7.47 (4.53–11.05)	2.44 (1.00–4.25)	2.11 (0.95–3.49)
Case-detection ratio	83% (75–91%)	90% (85–95%)	76% (67–84%)	88% (79–95%)	95% (90–98%)	85% (75–94%)
Incidence rate in 2019, per 100,000	218 (198–240)	1882 (1791–2003)	2260 (2025–2542)	136 (121–155)	1712 (1604–1831)	1921 (1721–2177)

μ: background mortality based on the World Population Prospects, 2016 for Kenya and 2019 for Blantyre (Malawi)

HIV-related mortality: AIDS-related deaths/PLHIV for aged 15 and above from the UNAIDS data

5% per month (0.61 per year): an extreme case for gold miner in pre-ART era [26]

### Sensitivity analyses

Across all mortality scenarios considered (Additional file 1-Section E), differences in duration were around one month, except for Lao PDR. The highest levels of assumed mortality led to up to a 7 percentage-point lower case-detection ratio than for the lowest assumed mortality. Sensitivity analysis assuming an extremely high excess TB mortality for PLHIV (Table 3) led to up to 15 percentage-point lower CDRs and correspondingly higher incidence rates. However, for both Kenya and Blantyre, the case-detection ratios were still not lower than that of HIV negative populations. Considering a broader range of symptoms resulted in shorter durations of the asymptomatic phase but longer time-spent on care-seeking given the TB prevalence survey and notification data. Estimates of rates of progression to symptoms were systematic under-estimates in the presence of symptom reversion, with an error that depended roughly linearly on reversion rate (with gradient of prevalent symptomatic TB over asymptomatic TB). The time asymptomatic and time symptomatic were both systematic over-estimates with symptom reversion.

## Discussion

By calibrating simple models of TB disease and care-seeking progression to prevalence survey and notification data, we were able to infer the typical duration of asymptomatic bacteriologically-positive TB to be around six months. However, there is a large variation between settings, with longer durations of asymptomatic disease in the included Asian settings. The asymptomatic phase typically comprised around half the total time before notification. For countries that reported care-seeking history in their prevalence surveys, we were able to estimate the average timing of initial care-seeking, finding this was approximately halfway between becoming symptomatic and ultimate diagnosis. We found limited evidence of age-dependence in overall durations, with a hint of longer durations in older age groups, but did find substantially shorter disease durations in people living with HIV. Our analysis of TB care-seeking, diagnosis and treatment outcome cascades also showed substantial differences between settings, and meaningful losses before the first symptom developed and care-seeking. Taken together, these findings suggest that important opportunities exist to identify people with tuberculosis earlier in their disease course through screening and community-based active case finding interventions, potentially improving individual outcomes and reducing transmission.

TB disease prior to care-seeking, including the asymptomatic phase, is beyond the reach of passive case-finding: improvements in diagnosis and retention at the clinic will not shorten these delays nor avert deaths during this phase. Cases that are truly asymptomatic can only be found by active approaches to screening based on exposure, chest X-ray or other novel rule-in tests - based in either the community or clinics. The symptomatic period prior to care seeking would be amenable to intervention by symptom-based screening approaches and potentially health-messaging or improved access to care. Understanding the duration of these phases and their contributions to transmission and mortality is therefore key to understanding the relative potential benefits of more active approaches to finding cases [26]. Although, between 25% and 50% of the total duration (and likely a higher proportion of transmission and pre-treatment mortality) occurs once those with TB have started seeking care, when improvements in passive case-finding and reductions in pre-treatment loss-to-follow-up (which has been observed a 13% in Asia and 18% in African settings) can help [28]. While there is considerable consistency between settings in our results, the reasons for the longer durations of asymptomatic disease in Asian settings are not clear, and seem not to relate to HIV prevalence. These may include cultural factors around recognition of disease or willingness to report it, differences in strains, or differences in cough associated with smoking or air quality which may mask TB symptoms. It is noteworthy that these settings also have longer total durations of TB disease. Future work could include the development of hierarchical models, which would pool data across countries and potentially smooth out some of these differences.

Our method also generates estimates of TB incidence: using literature estimates of TB disease mortality and self-cure rates, we inferred the proportions that fail to reach notification. Our incidence estimates are similar to those of WHO [1], which is not surprising since these are based on the same data. Our estimates have lower uncertainty, which may reflect under-estimation of uncertainty by our approach in not including uncertainty in TB mortality rates.

Our approach has quantified aspects of TB natural history that are potentially important but poorly characterised, relying on historical or anecdotal data. For example, mathematical models of TB transmission have from the outset often included progression in smear status [25]. In an analysis of cross-sectional data, this smear conversion rate is confounded with the proportion of individuals who are smear-positive from very early in their disease. Our posterior shows a high correlation between these parameters, but the gradient of initial smear-positivity with respect to conversion rate is broadly consistent between settings. Our pooled estimate should be useful for modellers wishing to parametrize joint uncertainty around these features.

There has been an increased realisation that TB disease is less dichotomous and more dynamic than conventional paradigms have allowed [8]. Our findings complement work in this area by describing the dynamics through asymptomatic disease and different levels of infectiousness while symptomatic. (We have used the term asymptomatic TB as synonymous with the term sub-clinical TB used in some discussions.) The substantial fraction of time spent asymptomatic means there is the potential for this phase to make important contributions to TB transmission. The key unknown factor here is the relative infectiousness of the asymptomatic phase; without this quantity, we are not able to estimate this contribution. While some evidence suggests that the natural history of TB disease may include progression and remission [8], we have assumed a one-way progression through the asymptomatic phase, smear status, and care-seeking - we were limited here by our reliance on cross-sectional data. However, our transitions between these cruder states could be considered as characterising the net average transitions in a population experiencing more complex dynamics among sub-states. This means that our estimated durations are best interpreted as mean time spent in each state and that proportions developing symptoms (e.g., Fig. 4) require caveat: with symptom reversion, the proportion ever reaching symptoms will be higher. Our sensitivity analysis, fitting our model to simulated data from models that include reversion as well as progression, explores these effects (see Additional file 1 section E3). More data constraining symptom dynamics would be necessary to meaningfully include additional complexity around this in our inferential approach. Including questions in TB prevalence surveys to distinguish current and historical symptoms and care could help quantify intermittency. Similarly, there may be heterogeneity in disease-course, which we were not able to consider. For example, there may be a mixture of TB disease types with differing symptom trajectories or rates of progression.

HIV-associated TB has long been known to have a more rapid TB disease progression [10], but data to quantify this has been limited [29], and most models and estimates of TB burden assume the same case detection ratio in PLHIV as among HIV-negative people [30]. We were able to provide the first empirical estimate of this quantity. Our sensitivity analysis considered from zero excess TB/HIV mortality through to an extremely high level and consistently found TB case-detection ratios for PLHIV that were higher than for HIV-negative people. We were not able to stratify by ART status, but increases in ART coverage and regular healthcare visits for people on ART may have helped improve TB case detection among PLHIV. We found an asymptomatic TB disease duration shorter than five months in people living with HIV, and the delays from the first symptom developed to case-detection also much shorter than those for HIV-negative people. The duration of TB disease in PLHIV has been suggested to decrease with CD4 cell count [31]; our results are consistent with this, but lack of CD4 data meant was not able to test this hypothesis. For those countries with high HIV-prevalence that lacked HIV-stratified data, our durations are expected to be lower than those experienced by HIV-negative people.

Comparing our model estimates of the delays from TB onset to notification with empirical estimates from P:N ratios, we find that empirical estimates systematically overestimate duration. This is because the empirical estimator implicitly assumes that all prevalent TB ends in notification, whereas in the model (and reality) self-cure and death are possible outcomes [32]. These unobserved competing events add to the rate at which individuals cease being prevalent, thus shortening the prevalent duration. The discrepancy between empirical and model estimates is largest where the CDR is lowest. Under-notification, e.g. in settings with large private sectors can further overestimate durations.

The duration of symptomatic TB is necessarily shorter than the total duration. Empirical estimates of TB duration based on TB prevalence surveys that used symptoms as an entry point to bacteriological testing [33] may therefore be biassed. Where symptoms alone are used to rule in, the empirical P:N ratio is measuring the duration of the symptomatic phase rather than the total duration of TB disease. The range of symptoms used can also affect estimated durations, with broader definitions shortening the duration of the asymptomatic phase. All the surveys we used either applied chest X-ray OR symptoms to rule-in for bacteriological testing while most of them considered only cough of ≥ 2 weeks and some included more symptoms (e.g. Tanzania used five symptoms [18]) for the symptom screening.

A limitation of our approach is that it relies on self-report for defining whether individuals have symptoms or have begun seeking care. Stigma, fear of diagnosis, or expectations of treatment may all affect and influence participants' willingness to report symptoms, even if they are recognised; recognition of symptoms may itself be influenced by cultural and epidemiological factors [34, 35]. Recall bias may play a role in limiting the accuracy of reports of care-seeking. We were limited in our ability to detect effects that may be real, such as age-dependency in durations, and to investigate smear status while asymptomatic, due to the relatively small numbers of TB cases found in prevalence surveys. Our projections of growth or decline rely solely on trends in notification data; where notification trends reflect changes in routine case finding performance, our projections of underlying incidence and prevalence trends may be incorrect. Finally, while most of our parameters have incorporated prior uncertainty, being treated as random variables in a Bayesian framework, we treated TB mortality and self-cure as fixed, which may underestimate uncertainty in our estimates.

A strength of our approach is that we have adopted a rigorous Bayesian framework, which includes uncertainty in inputs and outputs and could easily be generalised for use on other national or subnational data, and potentially developed as a software package. Unlike most approaches to P:N analyses, we do not make an assumption of equilibrium. Our assumption is the slightly more general and realistic constant rate of decline, which is an additional output.

## Conclusion

Active approaches to TB screening and case-finding should be considered in high TB burden settings, where up to 4 to 12 months with the infectious bacteriologically positive disease is spent without symptoms, and a comparable time again with symptoms before care-seeking initiation.

## Supplementary Information

**Additional file 1.** Supplementary materials including: Sources of data; Model details; Inference on delay and duration, posterior distribution by country sensitivity analysis

**Additional file 2.** TB prevalence survey characteristics

**Additional file 3.** Extracted data, 11 included settings

**Additional file 4.** Extracted data, Blantyre Malawi

**Additional file 5.** Extracted data, Kenya. TB prevalence data were from Enos et al.

## Data Availability

The individual-level datasets of the Blantyre data generated and/or analysed during the current study are not publicly available to avoid potential deductive disclosure. The aggregate datasets supporting the conclusions of this article are available in the TimeWz667/AsymTB repository, https://github.com/TimeWz667/AsymTB.git, and Additional files 2, 3, 4 and 5

## Abbreviations

CDR: Case detection ratio
CrIs: Credible intervals
MCMC: Markov chain Monte Carlo
PLHIV: People living with HIV
RR: Risk ratio
TB: Tuberculosis
WHO: World Health Organization
WPP: World Population Prospect
