# Supplementary material for: Durations of asymptomatic, symptomatic, and care-seeking phases of tuberculosis disease with a Bayesian analysis of prevalence survey and notification data
Source: BMC Med. 2021 Nov 10;19:298. doi: 10.1186/s12916-021-02128-9 (PMC8579670; doi:10.1186/s12916-021-02128-9)
Supplement: Supplementary file 1 — Additional file 1. Supplementary materials including: Sources of data; Model details; Inference on delay and duration, posterior distribution by country sensitivity analysis [file 12916_2021_2128_MOESM1_ESM.pdf]

Supplementary materials

Contents

A Models **3**
A.1 Notations and general assumptions . . . 3
A.1.1 Notations . . . 3
A.1.2 States . . . 3
A.1.3 Parameters . . . 4
A.1.4 Steady-declining condition . . . 4
A.2 Model A: Asymptomatic/Symptomatic TB . . . 6
A.3 Model B: Asymptomatic/Symptomatic TB considering care-seeking intentions . . . 8
A.4 Model C: Asymptomatic/Symptomatic TB considering smear types . . . 10
A.5 Empirical model . . . 12
A.5.1 Model . . . 12
A.5.2 Software and sampling details . . . 13

B Data description and posterior parameters by country **14**
B.1 Data description . . . 14
B.2 Cambodia (KHM) . . . 15
B.2.1 Data and model estimates . . . 15
B.2.2 Posterior parameters . . . 16
B.3 Ethiopia (ETH) . . . 18
B.3.1 Data and model estimates . . . 18
B.3.2 Posterior parameters . . . 19
B.4 Kenya (KEN) . . . 21
B.4.1 Data and model estimates . . . 21
B.4.2 Posterior parameters . . . 22
B.5 Lao PDR (LAO) . . . 24
B.5.1 Data and model estimates . . . 24
B.5.2 Posterior parameters . . . 25
B.6 Malawi (MWI) . . . 27
B.6.1 Data and model estimates . . . 27
B.6.2 Posterior parameters . . . 28
B.7 Pakistan (PAK) . . . 30
B.7.1 Data and model estimates . . . 30
B.7.2 Posterior parameters . . . 31
B.8 Philippines (PHL) . . . 33
B.8.1 Data and model estimates . . . 33

B.8.2 Posterior parameters . . . 34
B.9 Uganda (UGA) . . . 36
B.9.1 Data and model estimates . . . 36
B.9.2 Posterior parameters . . . 37
B.10 Tanzania (TZA) . . . 39
B.10.1 Data and model estimates . . . 39
B.10.2 Posterior parameters . . . 40
B.11 Vietnam (VNM) . . . 42
B.11.1 Data and model estimates . . . 42
B.11.2 Posterior parameters . . . 43
B.12 Zambia (ZMB) . . . 45
B.12.1 Data and model estimates . . . 45
B.12.2 Posterior parameters . . . 46

C Inference: epidemiological indices **48**

D Inference: delay and healthcare cascade **51**
D.1 Estimation of delay and duration . . . 51

E Inference: sensitivity analyses **53**
E.1 Mortality . . . 53
E.2 Symptomatic definition . . . 55
E.3 Symptom reversion rate sensitivity analyses . . . 56
E.3.1 Theoretical model . . . 56
E.3.2 Simulation study . . . 58

A Models

A.1 Notations and general assumptions

We construct our models as state space models using ordinary differential equations (ODE). Different model variants are employed depending on the data available. All transitions among states apply constant transition rates (Markovian assumption). We solve the models subject to a steady-state condition (described below). Based on the solution, we then develop statistical models fitting to data with a Bayesian framework to estimate free parameters. Finally, we use the posterior parameters to calculate the duration of asymptomatic TB and the delay to case-detection from symptomatic onset.

A.1.1 Notations

N_s Number of active TB in state s

N Number of all active TB ($= \sum_s N_s$)

X_s Proportion of active TB in state s among all active TB ($= N_s/N$)

A.1.2 States

Active TB in this study matches the standard definition of bacteriologically confirmed cases in TB prevalence surveys. For different model variants, we focus on different ways to define the states of active TB. Ideally, all active TB could be detected by an active case-finding programme with a sensitive screening algorithm. However, in this modelling, we assume all TB case notifications are from passive case-finding (i.e. autonomous care-seeking). There are two main states of asymptomatic TB (A) and symptomatic TB (S) in the general case. The end points of care-seeking aligns with case notification. The operational features are as follows.

	Asymptomatic TB (A)	Symptomatic TB (S)
TB-related symptoms	No	Yes
Care-seeking intention	No	Yes
TB-related mortality	No	Yes
Sputum smear microscopy	Usually low precision	Positive / Negative

A.1.3 Parameters

The free parameters are those treated as random variables and inferred through calibration, or parameters derived from other free parameters. Others parameters were sourced from external data or assumptions.

Notation	Description	Distribution/Constant
Λ	incident cases of asymptomatic TB	Distribution
θ	symptom development rate	Distribution
π	proportion of smear-positive at symptom onset	Distribution
τ	conversion rate, from smear-negative to -positive	Distribution
ϕ	annual decline rate for incidence	Distribution
ν	self-cure rate	Distribution
η	presence rate of care-seeking intentions	Distribution
ρ_s	care-seeking rate of state s	Distribution
μ	background death rate	Data
μ_s	untreated death rate of state s	Data

A.1.4 Steady-declining condition

We assumed constant decreasing rates of prevalent cases for all settings during the study period. In a stationary condition. That is,

$$\frac{\mathrm{d}N_s/\mathrm{d}t}{N_s} = \frac{\mathrm{d}N/\mathrm{d}t}{N} = -\phi \qquad \forall s \in \mathit{states}$$

Inference 1 The steady-declining condition hold only if the proportion of every active TB state is in an equilibrium.

$$\begin{aligned}
\frac{\mathrm{d}N_s}{\mathrm{d}t} + \phi N_s &= \frac{\mathrm{d}NX_s}{\mathrm{d}t} + \phi N_s \\
&= \left(X_s\frac{\mathrm{d}N}{\mathrm{d}t} + N\frac{\mathrm{d}X_s}{\mathrm{d}t}\right) + \phi N_s \\
&= \left(X_s(-\phi N) + N\frac{\mathrm{d}X_s}{\mathrm{d}t}\right) + \phi N_s \\
&= -\phi N_s + N\frac{\mathrm{d}X_s}{\mathrm{d}t} + \phi N_s \\
&= N\frac{\mathrm{d}X_s}{\mathrm{d}t} \qquad \forall s \in \mathit{states}
\end{aligned}$$

Therefore, for all state s, $\frac{\mathrm{d}N_s}{\mathrm{d}t} + \phi N_s = 0$ when $\frac{\mathrm{d}X_s}{\mathrm{d}t} = 0$

Inference 2 The case notification of each state which have care-seeking intention is declining with the same decline rate (ϕ).

$$
\begin{aligned}
Notification_s &= \rho_s N_s \\
log(Notification_s) &= log(\rho_s) + log(N_s) \\
\frac{\mathrm{d}Notification_s/\mathrm{d}t}{Notification_s} &= \frac{\mathrm{d}N_s/\mathrm{d}t}{N_s} && time\,derivative \\
&= -\phi && \forall s \in states\,with\,care\text{-}seeking\,intention
\end{aligned}
$$

Inference 3 For asymptomatic TB (A), the incidence is proportional to the number of A in order to ensure the steady-declining condition in N_A. Therefore the incident case is declining with the same decline rate (ϕ) as well.

Duration and delay As we applied Markovian assumption for the models (compatible with ODE system with constant rates and continuous-time Markov chain), we can calculate the duration of asymptomatic TB and the delay to case-detection from symptomatic onset with Exponential distributions. See Appendix D.1.

A.2 Model A: Asymptomatic/Symptomatic TB

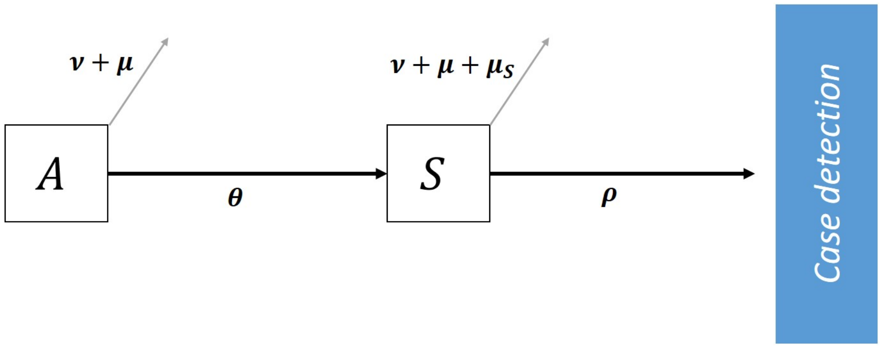

Figure 1: Model A

Model A models the two basic states: asymptomatic TB A and symptomatic S with following ordinary differential equations. State A will transition to State S once symptom onset. State S have motivations in care-seeking.

$$\frac{\mathrm{d}N_A}{\mathrm{d}t} = \Lambda - (\theta + \gamma_A)N_A$$
$$\frac{\mathrm{d}N_S}{\mathrm{d}t} = \theta N_A - (\rho_S + \gamma_S)N_S$$

where

$$\gamma_A = \mu + \nu$$
$$\gamma_S = \mu + \mu_S + \nu$$

Steady declining condition The system can be written as

$$\frac{\mathrm{d}N_A}{\mathrm{d}t} = \Lambda - (\theta + \gamma_A)N_A = -\phi N_A \quad (1)$$
$$\frac{\mathrm{d}N_S}{\mathrm{d}t} = \theta N_A - (\rho_S + \gamma_S)N_S = -\phi N_S \quad (2)$$
$$N_A + N_S = N \quad (3)$$

By Equation 2, the ratios of prevalent cases are

$$N_A : N_S = (\rho_S + \gamma_S - \phi) : \theta$$

Therefore, given the size of total active TB N,

- Prevalent A: $N_A = N \times (\rho_S + \gamma_S - \phi)/(\rho_S + \gamma_S - \phi + \theta)$
- Prevalent S: $N_S = N \times \theta/(\rho_S + \gamma_S - \phi + \theta)$
- Notification: $= \rho_S N_S$
- Incident cases: $\Lambda = (\theta + \gamma_A - \phi)N_A$

Note that prevalence, case notification, and incident cases are linearly related, so they follow the same declining rate. For example,

$$\begin{aligned}
\Lambda &= (\theta + \gamma_A - \phi) \times N_A && \textit{Incidence and Prevalence} \\
\log(\Lambda) &= \log(\theta + \gamma_A - \phi) + \log(N_A) \\
\frac{1}{\Lambda}\frac{\mathrm{d}\Lambda}{\mathrm{d}t} &= \frac{1}{N_A}\frac{\mathrm{d}N_A}{\mathrm{d}t} && \textit{Take the time derivative} \\
&= -\phi
\end{aligned}$$

Inference: duration and delay As we assume exponentially-distributed progression delays (in line with the assumption of ODE models). The duration of a state is operationally defined as the mean time to any next state. The delay to case detection is defined as the time to next event given that the next event event is case-detection.

$$\begin{aligned}
Duration_A &= E_A(T) \\
&= \frac{1}{\theta + \gamma_A} \\
Duration_S &= E_S(T|case\,detection) \\
&= E_S(T) && \textit{See Appendix D.1} \\
&= \frac{1}{\rho_S + \gamma_S} \\
Delay_S &= Duration_S
\end{aligned}$$

where T denotes time to next event, $E_s(.)$ is expectation function of time stay in state s, and $E_s(.|event)$ is expectation function of waiting time toward *event* in state s. See Appendix D.1 for the detailed derivation.

A.3 Model B: Asymptomatic/Symptomatic TB considering care-seeking intentions

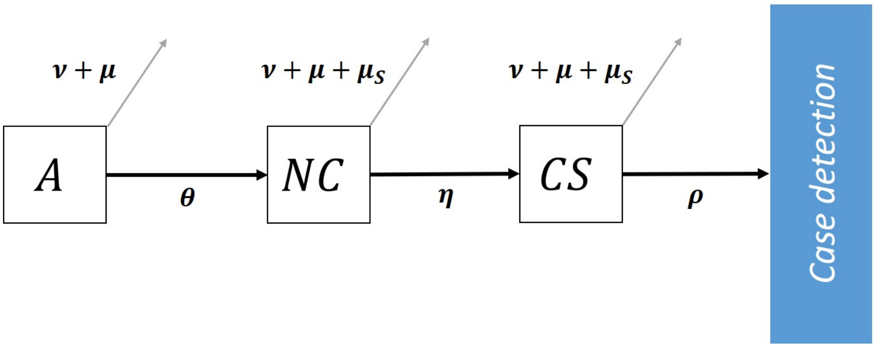

Figure 2: Model B

Model B divides S into symptomatic TB without care-seeking intentions (NC) and with care-seeking intentions (CS). Only CS can be notified under passive case-finding.

$$\begin{aligned}\frac{\mathrm{d}N_A}{\mathrm{d}t} &= \Lambda - \theta N_A - \gamma_A N_A \\ \frac{\mathrm{d}N_{NC}}{\mathrm{d}t} &= \theta N_A - (\eta + \gamma_{NC})N_{NC} \\ \frac{\mathrm{d}N_{CS}}{\mathrm{d}t} &= \eta N_{NC} - (\rho_{CS} + \gamma_{CS})N_{CS}\end{aligned}$$

Where

$$\begin{aligned}\gamma_A &= \mu + \nu \\ \gamma_{NC} &= \mu + \mu_{NC} + \nu \\ \gamma_{CS} &= \mu + \mu_{CS} + \nu\end{aligned}$$

Steady declining condition The system can be written as

$$\frac{\mathrm{d}N_A}{\mathrm{d}t} = \Lambda - \theta N_A - \gamma_A N_A = -\phi N_A \tag{4}$$

$$\frac{\mathrm{d}N_{NC}}{\mathrm{d}t} = \theta N_A - (\eta + \gamma_{NC})N_{NC} = -\phi N_{NC} \tag{5}$$

$$\frac{\mathrm{d}N_{CS}}{\mathrm{d}t} = \eta N_{NC} - (\rho_{CS} + \gamma_{CS})N_{CS} = -\phi N_{CS} \tag{6}$$

$$N_A + N_{NC} + N_{CS} = N \tag{7}$$

By Equation 5 and Equation 6, the ratios of prevalent cases are

$$N_A : N_{NC} : N_{CS} = \frac{\eta + \gamma_{NC} - \phi}{\theta} : 1 : \frac{\eta}{\rho_{CS} + \gamma_{CS} - \phi}$$

We can rescale the above ratios by $\frac{\eta+\gamma_{NC}-\phi}{\theta} + 1 + \frac{\eta}{\rho_{CS}+\gamma_{CS}-\phi}$ to calculate $X_A : X_{NC} : X_{CS}$

Therefore, given the size of total active TB, N,

- Prevalent A: $N_A = N \times X_A$
- Prevalent NC: $N_{NC} = N \times X_{NC}$
- Prevalent CS: $N_{CS} = N \times X_{CS}$
- Notification: $= \rho_{CS} N_{CS}$
- Incident cases: $\Lambda = (\theta + \gamma_A - \phi) N_A$

Inference: duration and delay

$$\begin{aligned}
Duration_A &= \frac{1}{\theta + \gamma_A} \\
Duration_{NC} &= \frac{1}{\eta + \gamma_{NC}} \\
Duration_{CS} &= \frac{1}{\rho_{CS} + \gamma_{CS}} \\
Delay_{CS} &= Duration_{CS}
\end{aligned}$$

A.4 Model C: Asymptomatic/Symptomatic TB considering smear types

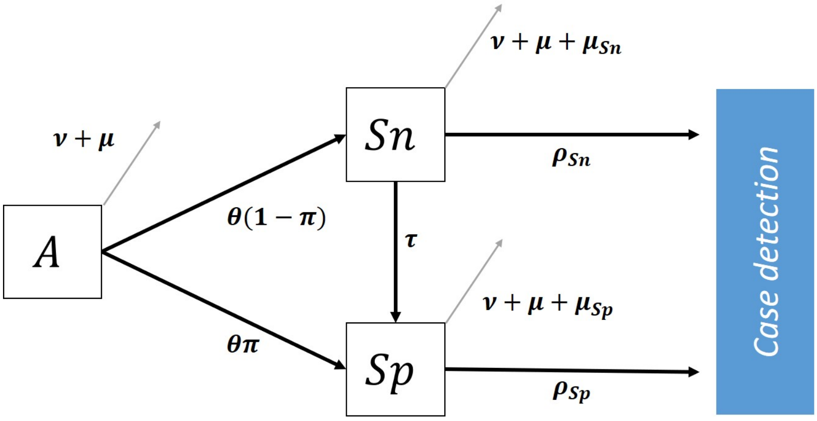

Figure 3: Model C

Model C divides S into symptomatic smear-negative TB (Sn) and symptomatic smear-positive TB (Sp) as following ordinary differential equations. A can develop to both Sn and Sp, and Sn can convert to Sp. Both Sn and Sp have care-seeking intentions.

$$\frac{\mathrm{d}N_A}{\mathrm{d}t} = \Lambda - \theta N_A - \gamma_A N_A$$
$$\frac{\mathrm{d}N_{Sn}}{\mathrm{d}t} = (1-\pi)\theta N_A - \tau N_{Sn} - (\rho_{Sn} + \gamma_{Sn})N_{Sn}$$
$$\frac{\mathrm{d}N_{Sp}}{\mathrm{d}t} = \pi\theta X_A + \tau X_{Sn} - (\rho_{Sp} + \gamma_{Sp})N_{Sp}$$

Where

$$\gamma_A = \mu + \nu$$
$$\gamma_{Sn} = \mu + \mu_{Sn} + \nu$$
$$\gamma_{Sp} = \mu + \mu_{Sp} + \nu$$

Steady declining condition Under the condition, the system can be written as

$$\frac{\mathrm{d}N_A}{\mathrm{d}t} = \Lambda - \theta N_A - \gamma_A N_A = -\phi N_A \tag{8}$$
$$\frac{\mathrm{d}N_{Sn}}{\mathrm{d}t} = (1-\pi)\theta N_A - \tau N_{Sn} - (\rho_{Sn} + \gamma_{Sn})N_{Sn} = -\phi N_{Sn} \tag{9}$$
$$\frac{\mathrm{d}N_{Sp}}{\mathrm{d}t} = \pi\theta N_A + \tau N_{Sn} - (\rho_{Sp} + \gamma_{Sp})N_{Sp} = -\phi N_{Sp} \tag{10}$$
$$N_A + N_{NC} + N_{CS} = N \tag{11}$$

By Equation 9,

$$N_{Sn} = \frac{(1-\pi)\theta}{\tau + \rho_{Sn} + \gamma_{Sn} - \phi} N_A$$

With Equation 10,

$$N_{Sp} = \left(\pi + \frac{(1-\pi)\tau}{\tau + \rho_{Sn} + \gamma_{Sn} - \phi}\right)\frac{\theta}{\rho_{Sn} + \gamma_{Sn} - \phi}N_A$$

Therefore, given the size of total active TB, N and the above ratios,

- Prevalent A: $N_A = N/\left(1 + \frac{(1-\pi)\theta}{\tau+\rho_{Sn}+\gamma_{Sn}-\phi} + \left(\pi + \frac{(1-\pi)\tau}{\tau+\rho_{Sn}+\gamma_{Sn}-\phi}\right)\frac{\theta}{\rho_{Sn}+\gamma_{Sn}-\phi}\right)$
- Prevalent Sn: $N_{Sn} = \frac{(1-\pi)\theta}{\tau+\rho_{Sn}+\gamma_{Sn}-\phi}N_A$
- Prevalent Sp: $N_{Sp} = \left(\pi + \frac{(1-\pi)\tau}{\tau+\rho_{Sn}+\gamma_{Sn}-\phi}\right)\frac{\theta}{\rho_{Sn}+\gamma_{Sn}-\phi}N_A$
- Notification, smear-negative: $\rho_{Sn}N_{Sn}$
- Notification, smear-positive: $\rho_{Sp}N_{Sp}$
- Incident cases, Λ: $(\theta + \gamma_A - \phi)N_A$

Inference: duration and delay

$$\begin{aligned}
Duration_A &= \frac{1}{\theta + \gamma_A} \\
Duration_{Sn} &= \frac{1}{\rho_{Sn} + \gamma_{Sn}} \\
Duration_{Sp} &= \frac{1}{\rho_{Sp} + \gamma_{Sp}} \\
Delay_{Sn} &= Duration_{Sn} + Duration_{Sp} \\
Delay_{Sp} &= Duration_{Sp}
\end{aligned}$$

A.5 Empirical model

A.5.1 Model

The empirical models were developed with ***stan*** and implemented with package ***rstan*** in ***R***. The model structure is as follows

Layer 1, Prior distribution: In general, parameters of rates are sample from inverse-gamma distribution of $(shape = 1, scale = 1)$ otherwise stated. To be noted that some parameters only used in specific model variants. All rates are per capita-year rates. We choose $invGamma(1, 1)$, which implies the time-to a specific event follows a $Gamma(1, 1)$ with a one year in both values of mean and standard deviation. The prior distributions for the rates were chosen for at least covering $0 - 3$ years of time-to events

$$\begin{aligned}
\theta &\sim invGamma(1, 1) \\
\eta &\sim invGamma(1, 1) \\
\rho_s &\sim invGamma(1, 1) \\
\tau &\sim U(0, 0.5) \\
\pi &\sim U(0, 1) \\
\nu &\sim U(0.1, 0.3) \\
\phi &\sim U(-0.2, 0.2)
\end{aligned}$$

where $invGamma(a, b)$ is inverse-gamma distribution with shape of a and scale of b, and $U(a, b)$ is uniform distribution with lower-bound of a and upper-bound of b.

Layer 2, Prevalence survey:

$$\begin{aligned}
X_s &= f_s(.) \\
prev_s &= X_s N/M \\
Prevalence_s &\sim Binomial(prev_s, M) \\
&\forall s \in states
\end{aligned}$$

where $f_s(.)$ denotes the steady-state solution of state s, $prev_s$ is prevalence of state s in the model, $Prevalence_s$ is the prevalent cases matching state s in a prevalence survey, M is the number of surveyed subjects in a prevalence survey, and $Binomial(a, b)$ is Binomial distribution with probability of a and size of b. We neglect design effects.

Layer 3, Prevalence extrapolation: According to Appendix A.1.4, prevalence evolves with notification under the steady-decline condition.

$$\begin{aligned} prev_{s,t} &= prev_s \times e^{-\phi(t-t_0)} \\ &\forall s \in states \\ &\forall t \in years\ with\ notification\ data \end{aligned}$$

where t denotes year, t_0 for the year with the prevalence survey, and $prev_{s,t}$ is the prevalence of state s in year t.

Layer 4, Notification

$$\begin{aligned} Notification_{s,t} &\sim Poisson(\rho_s \times prev_{s,t} \times Population_t) \\ &\forall s \in states\ with\ care\text{-}seeking\ intentions \\ &\forall t \in years\ with\ notification\ data \end{aligned}$$

where $Notification_{s,t}$ denotes the notified cases of state s in year t, $Population_t$ is the population size in year t, ρ_s is the care-seeking rate of state s and $Poisson(a)$ is Poisson distribution with λ, event rate, of a.

A.5.2 Software and sampling details

The posterior distribution were sampled by Markov chain Monte Carlo with ***stan***'s default engine. We sampled three chains every model until the convergence in the last 3,000 samples defined as Gelman-Rubin convergence statistic (r hat) smaller than 1.05.

B Data description and posterior parameters by country

B.1 Data description

Table 1: Data for model fitting and mortality settings

Country	ISO	Data year		Background mortality per year		Excess TB mortality per year	
		Prevalence	Notification	Female	Male	asymptomatic	symptomatic
Cambodia	KHM	2011	2014–2019	0.0165	0.0187	0	0.0843
Ethiopia	ETH	2010	2015-2019	0.0058	0.0091	0	0.0709
Kenya	KEN	2016	2013–2019	0.0067	0.0093	0	0.0773
Lao PDR	LAO	2010	2013–2019	0.0161	0.0218	0	0.1066
Malawi	MWI	2013	2013–2019	0.0103	0.0165	0	0.0725
Pakistan	PAK	2010	2014–2019	0.0084	0.0119	0	0.0712
Philippines	PHL	2016	2015–2019	0.01	0.0159	0	0.066
Uganda	UGA	2014	2014–2019	0.0123	0.0176	0	0.0749
Tanzania	TZA	2012	2013–2019	0.0095	0.0124	0	0.0891
Vietnam	VNM	2017	2016–2019	0.0115	0.0153	0	0.0908
Zambia	ZMB	2013	2013–2019	0.0102	0.0132	0	0.0637

Background mortality: Background mortality rates used the average of the all cause mortality rate weighted by the WHO case-notification data by age and sex.

Excess mortality for symptomatic TB: summarised the mortality of 0.12 per year (70% for 10 years) for untreated smear-positive TB and 0.022 per year (20% for 10 years) for untreated smear-negative TB[1] by the proportion of smear-positive TB in case notification data.

To be noted that the model fitting were conducted with sex specific data while the results in the main text aggregated the results with the weights of estimated incident cases.

[1]Tiemersma EW, van der Werf MJ, Borgdorff MW, Williams BG, Nagelkerke NJ. Natural history of tuberculosis: duration and fatality of untreated pulmonary tuberculosis in HIV negative patients: a systematic review. PloS one. 2011 Apr 4;6(4):e17601.

B.2 Cambodia (KHM)

B.2.1 Data and model estimates

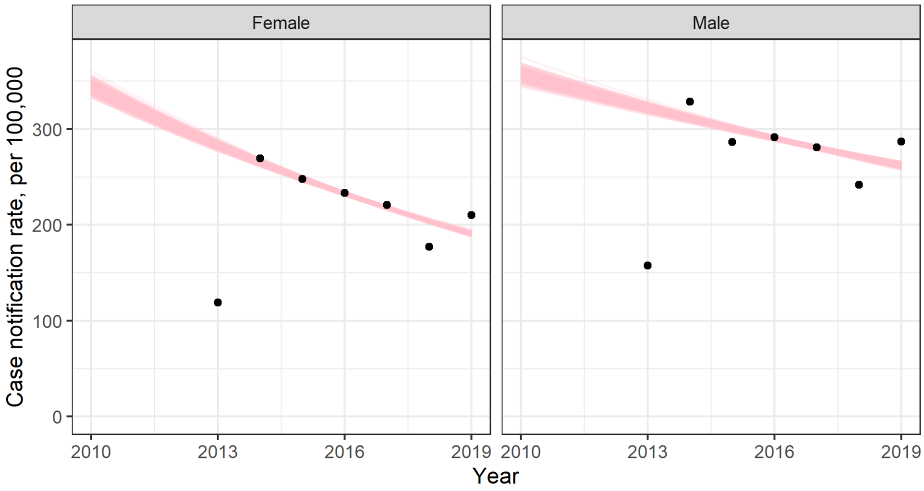

Figure 4: Case notification rate in model estimates and data by sex

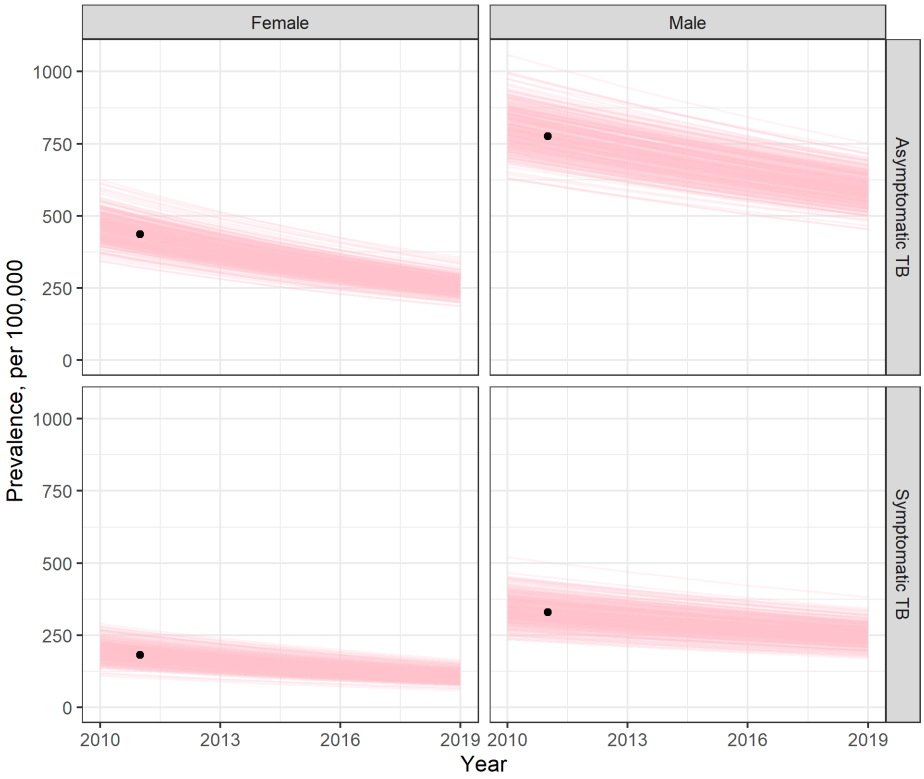

Figure 5: Prevalence in model estimates and data by sex and symptomatic or not

B.2.2 Posterior parameters

Table 2: Posterior distributions of parameters, Model B

Variable	Sex	Mean	SD	95% CrI	ESS	$\hat{R}$
rate to symptomatic onset	Female	0.85	0.09	(0.68, 1.04)	2236	0.999
rate to initial care-seeking	Female	24.46	17.36	(8.38, 66.85)	1941	1.000
rate to case-detection	Female	1.95	0.33	(1.39, 2.68)	2661	1.001
annual decline rate, %	Female	6.55	0.21	(6.14, 6.96)	3359	1.000
rate to symptomatic onset	Male	0.56	0.06	(0.46, 0.68)	2314	1.000
rate to initial care-seeking	Male	8.02	3.09	(4.20, 15.37)	2294	1.002
rate to case-detection	Male	1.30	0.19	(0.98, 1.73)	2580	1.000
annual decline rate, %	Male	3.46	0.20	(3.06, 3.84)	3454	1.000
self-cure rate	Shared	0.20	0.06	(0.10, 0.29)	2851	0.999

Table 3: Posterior distributions of parameters, Model C

Variable	Sex	Mean	SD	95% CrI	ESS	$\hat{R}$
rate to symptomatic onset	Female	0.85	0.10	(0.68, 1.05)	2358	1.001
rate to case-detection, smear-	Female	1.08	0.19	(0.78, 1.50)	1993	1.000
rate to case-detection, smear+	Female	2.46	0.61	(1.55, 3.91)	2763	1.000
annual decline rate, %	Female	6.55	0.22	(6.11, 6.97)	3171	1.001
rate to symptomatic onset	Male	0.56	0.05	(0.46, 0.68)	2398	1.001
rate to case-detection, smear-	Male	0.89	0.13	(0.66, 1.18)	1511	1.000
rate to case-detection, smear+	Male	1.34	0.26	(0.92, 1.92)	2472	1.000
annual decline rate, %	Male	3.46	0.20	(3.07, 3.84)	2867	1.000
smear conversion rate	Shared	0.78	0.58	(0.02, 1.88)	1061	1.001
smear+ at symptom onset	Shared	0.38	0.19	(0.03, 0.63)	1064	1.000
rate to self-cure	Shared	0.21	0.06	(0.11, 0.30)	2810	1.000

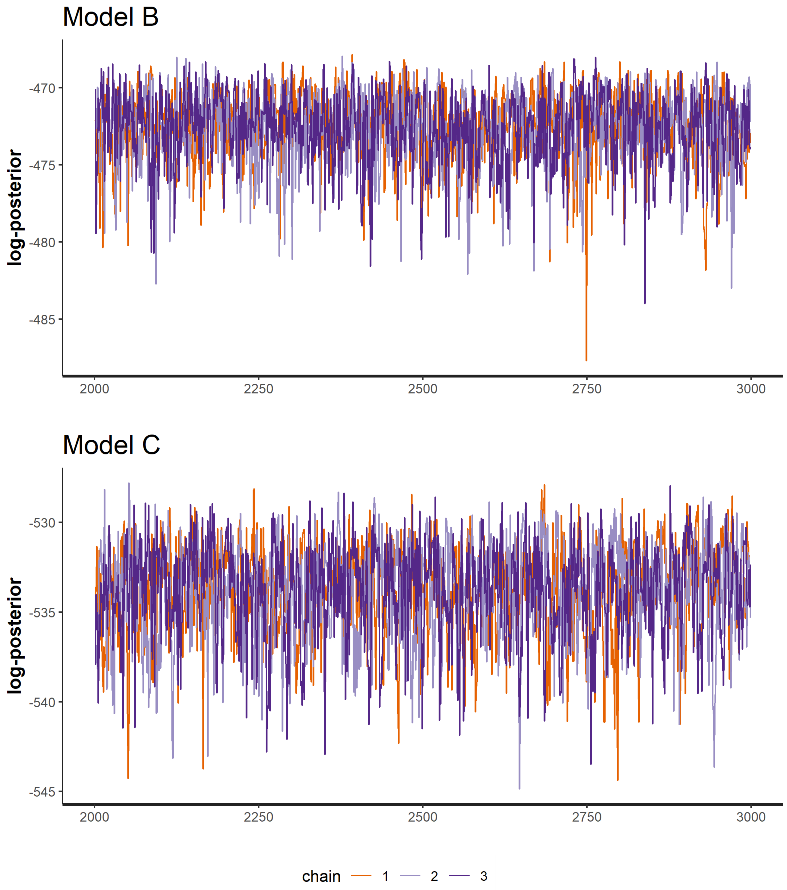

Figure 6: Trace of log-posterior probabilities

B.3 Ethiopia (ETH)

B.3.1 Data and model estimates

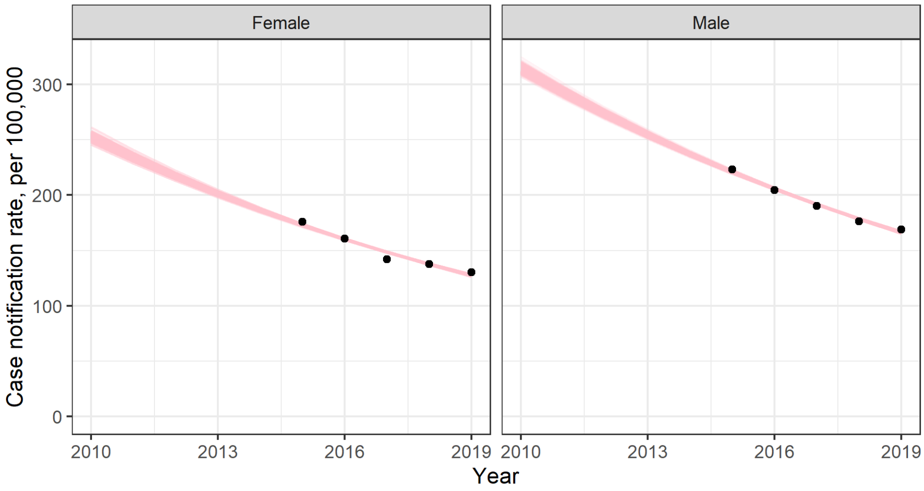

Figure 7: Case notification rate in model estimates and data by sex

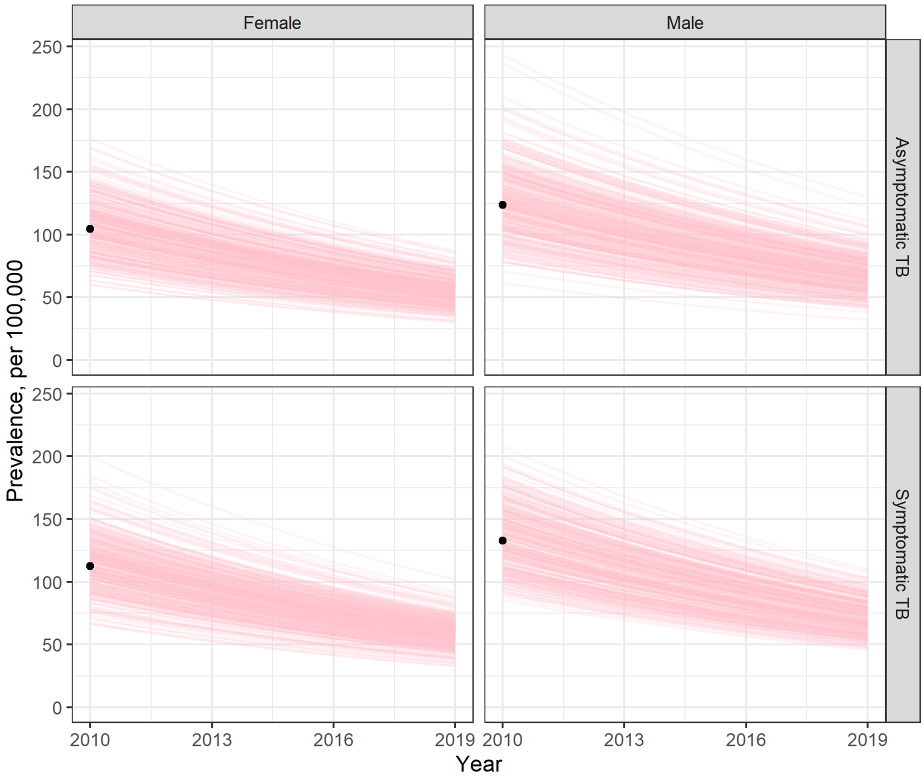

Figure 8: Prevalence in model estimates and data by sex and symptomatic or not

B.3.2 Posterior parameters

Table 4: Posterior distributions of parameters, Model A

Variable	Sex	Mean	SD	95% CrI	ESS	$\hat{R}$
rate to symptomatic onset	Female	2.64	0.51	(1.81, 3.81)	2433	0.999
rate to case-detection	Female	2.27	0.45	(1.54, 3.29)	2299	1.000
annual decline rate, %	Female	7.60	0.15	(7.31, 7.89)	4165	1.001
rate to symptomatic onset	Male	2.76	0.57	(1.87, 4.10)	2740	1.000
rate to case-detection	Male	2.36	0.45	(1.64, 3.41)	2644	1.000
annual decline rate, %	Male	7.08	0.13	(6.84, 7.34)	3462	1.000
self-cure rate	Shared	0.20	0.06	(0.10, 0.30)	4113	1.000

Table 5: Posterior distributions of parameters, Model C

Variable	Sex	Mean	SD	95% CrI	ESS	$\hat{R}$
rate to symptomatic onset	Female	2.61	0.51	(1.78, 3.74)	1645	1.000
rate to case-detection, smear-	Female	2.22	0.44	(1.54, 3.30)	980	1.001
rate to case-detection, smear+	Female	2.57	0.76	(1.46, 4.50)	1339	1.000
annual decline rate, %	Female	7.60	0.15	(7.31, 7.89)	2592	1.000
rate to symptomatic onset	Male	2.78	0.54	(1.91, 3.98)	1503	1.001
rate to case-detection, smear-	Male	2.21	0.43	(1.54, 3.22)	978	1.001
rate to case-detection, smear+	Male	2.41	0.62	(1.45, 3.88)	1143	1.000
annual decline rate, %	Male	7.08	0.13	(6.83, 7.34)	2077	1.000
smear conversion rate	Shared	0.79	0.59	(0.02, 1.89)	621	1.002
smear+ at symptom onset	Shared	0.34	0.13	(0.08, 0.51)	602	1.002
rate to self-cure	Shared	0.19	0.06	(0.10, 0.30)	2098	1.001

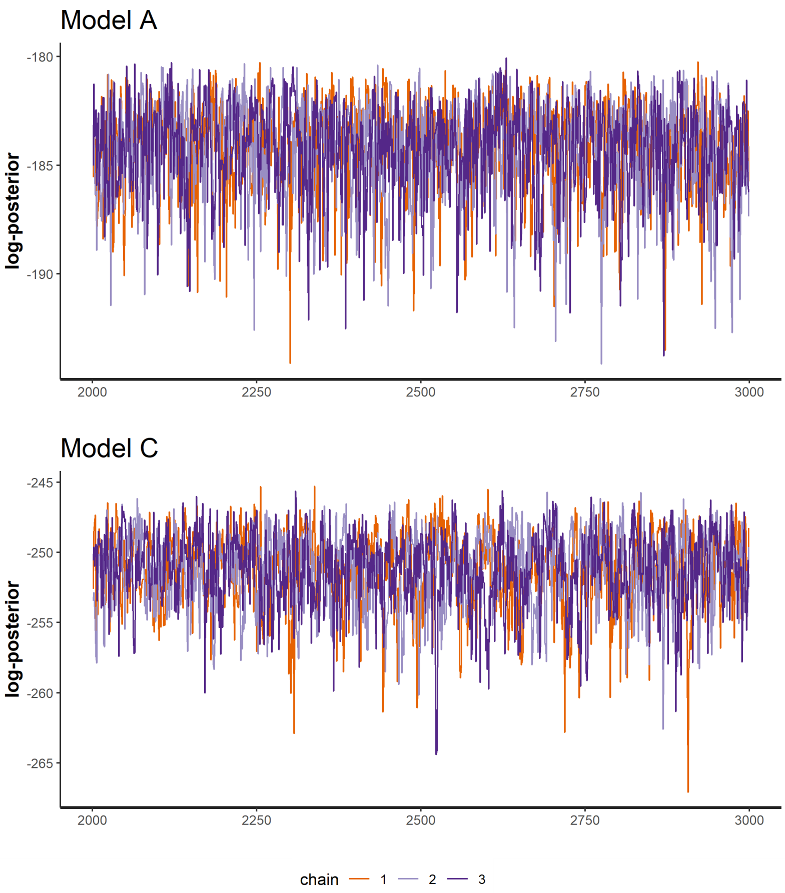

Figure 9: Trace of log-posterior probabilities

B.4 Kenya (KEN)

B.4.1 Data and model estimates

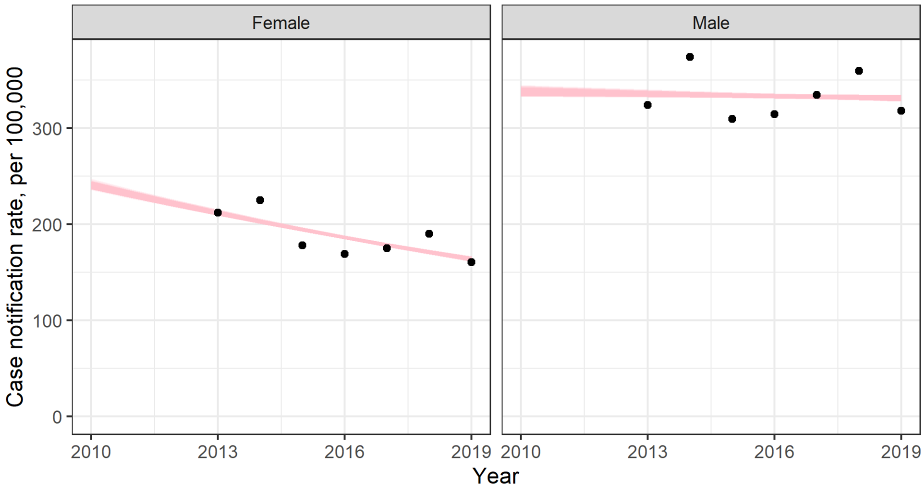

Figure 10: Case notification rate in model estimates and data by sex

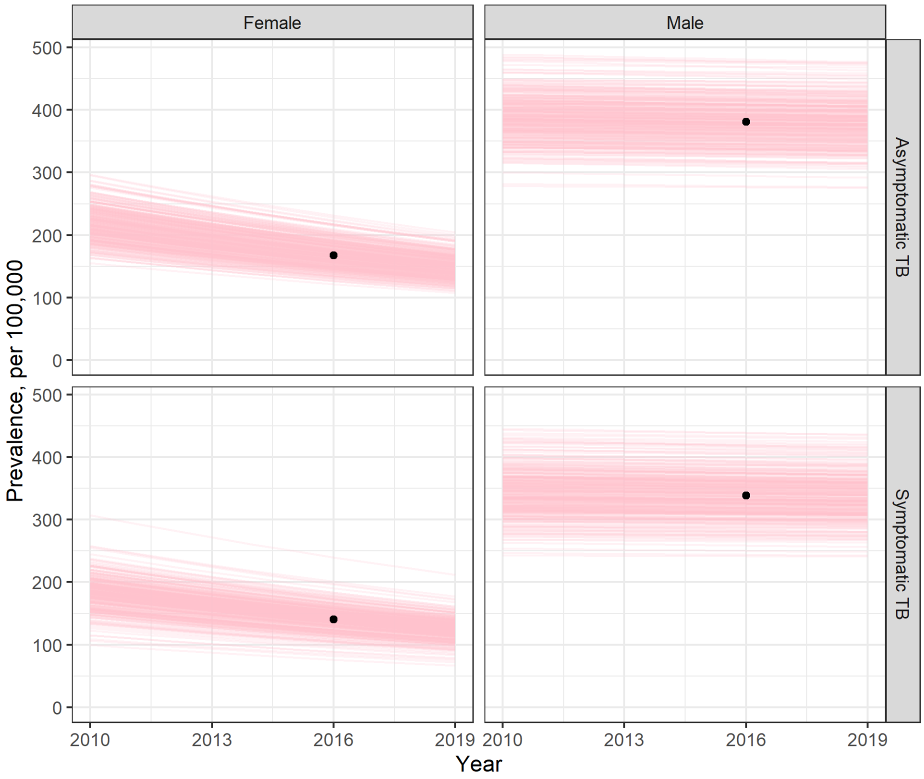

Figure 11: Prevalence in model estimates and data by sex and symptomatic or not

B.4.2 Posterior parameters

Table 6: Posterior distributions of parameters, Model B

Variable	Sex	Mean	SD	95% CrI	ESS	$\hat{R}$
rate to symptomatic onset	Female	1.32	0.18	(1.01, 1.73)	2205	1.001
rate to initial care-seeking	Female	2.40	0.44	(1.68, 3.37)	2680	1.000
rate to case-detection	Female	3.33	0.74	(2.19, 4.99)	2382	0.999
annual decline rate, %	Female	4.28	0.11	(4.06, 4.51)	3524	1.000
rate to symptomatic onset	Male	1.13	0.13	(0.91, 1.40)	2174	1.000
rate to initial care-seeking	Male	1.72	0.23	(1.33, 2.25)	2625	0.999
rate to case-detection	Male	2.70	0.47	(1.93, 3.77)	2913	1.000
annual decline rate, %	Male	0.22	0.09	(0.05, 0.39)	3828	1.000
self-cure rate	Shared	0.20	0.06	(0.10, 0.29)	2448	1.000

Table 7: Posterior distributions of parameters, Model C

Variable	Sex	Mean	SD	95% CrI	ESS	$\hat{R}$
rate to symptomatic onset	Female	1.31	0.17	(1.02, 1.69)	1911	1.001
rate to case-detection, smear-	Female	1.25	0.18	(0.96, 1.67)	1046	1.001
rate to case-detection, smear+	Female	1.53	0.31	(1.04, 2.24)	1819	1.000
annual decline rate, %	Female	4.28	0.11	(4.05, 4.50)	2587	1.000
rate to symptomatic onset	Male	1.14	0.13	(0.92, 1.41)	1686	1.000
rate to case-detection, smear-	Male	1.05	0.13	(0.82, 1.33)	1109	1.003
rate to case-detection, smear+	Male	0.93	0.13	(0.73, 1.21)	1742	1.000
annual decline rate, %	Male	0.22	0.08	(0.06, 0.39)	3323	0.999
smear conversion rate	Shared	0.96	0.52	(0.06, 1.87)	814	1.001
smear+ at symptom onset	Shared	0.28	0.16	(0.02, 0.56)	816	1.001
rate to self-cure	Shared	0.20	0.06	(0.10, 0.29)	2412	1.001

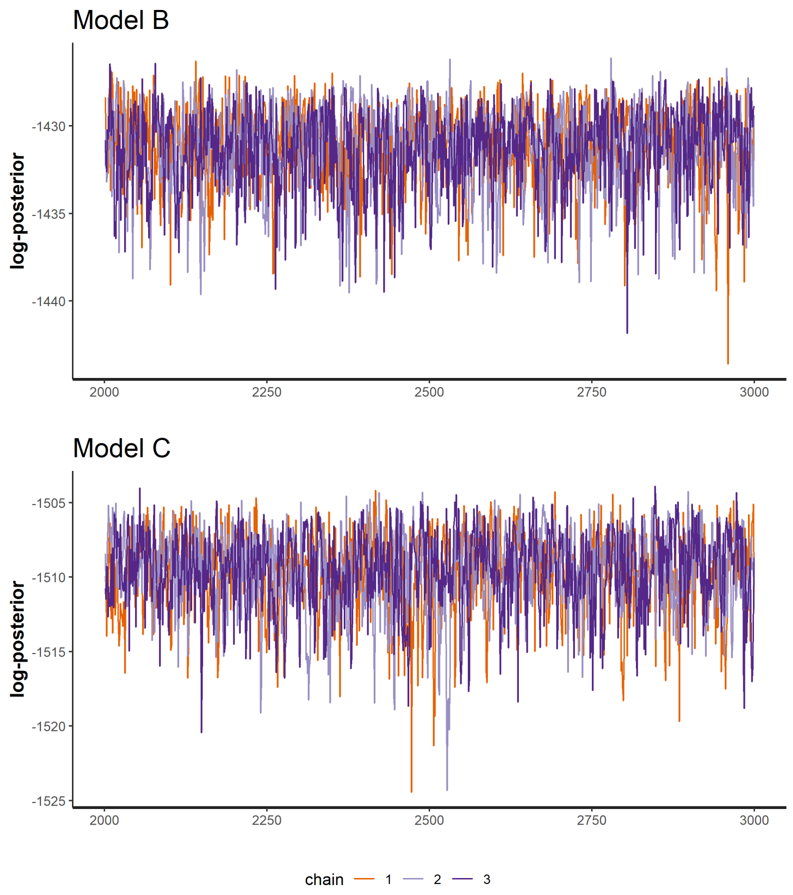

Figure 12: Trace of log-posterior probabilities

B.5 Lao PDR (LAO)

B.5.1 Data and model estimates

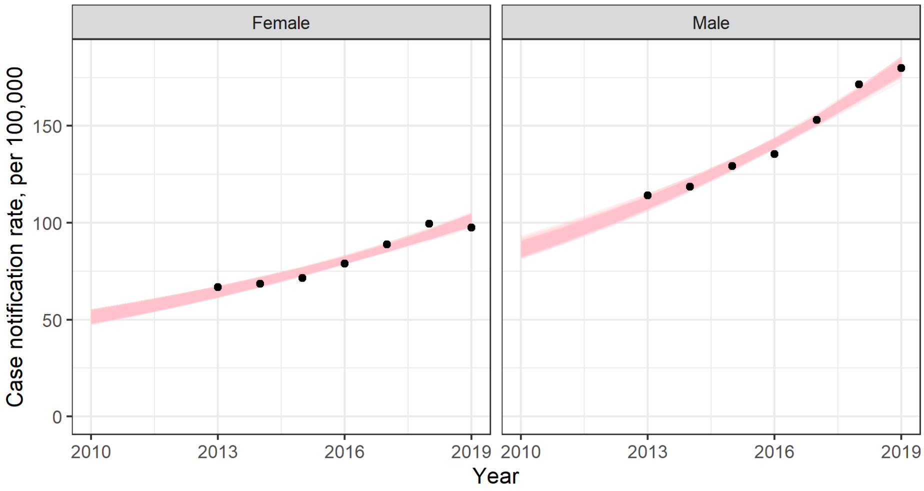

Figure 13: Case notification rate in model estimates and data by sex

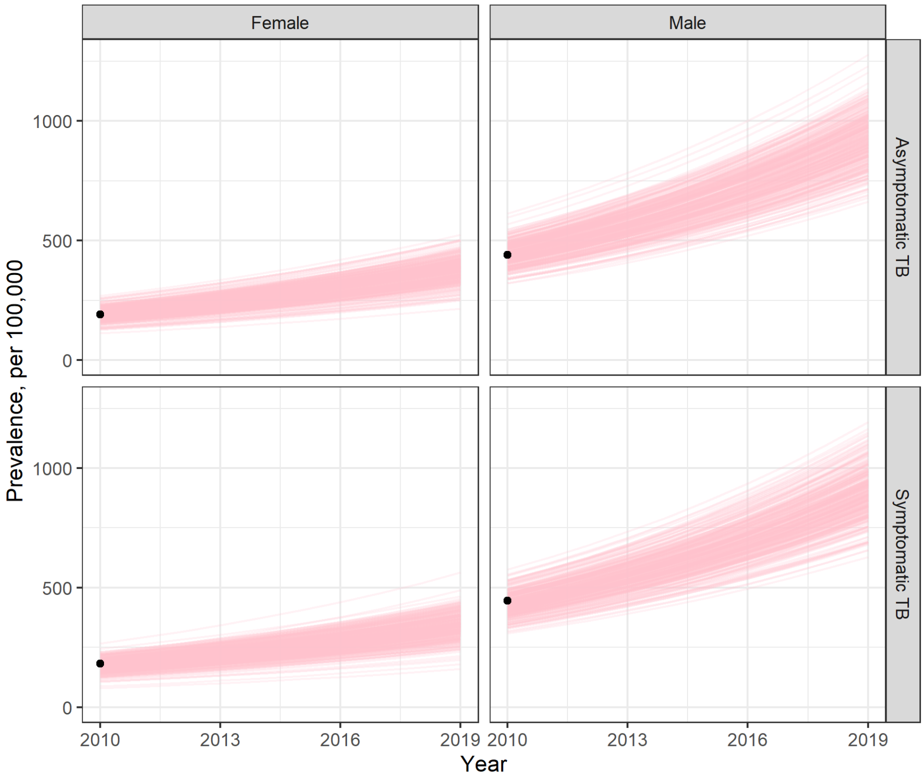

Figure 14: Prevalence in model estimates and data by sex and symptomatic or not

B.5.2 Posterior parameters

Table 8: Posterior distributions of parameters, Model A

Variable	Sex	Mean	SD	95% CrI	ESS	$\hat{R}$
rate to symptomatic onset	Female	0.65	0.13	(0.44, 0.96)	2541	1.000
rate to case-detection	Female	0.30	0.05	(0.22, 0.42)	2256	0.999
annual decline rate, %	Female	-7.50	0.44	(-8.34, -6.64)	3643	1.000
rate to symptomatic onset	Male	0.61	0.10	(0.44, 0.83)	2687	0.999
rate to case-detection	Male	0.20	0.02	(0.16, 0.26)	2908	1.000
annual decline rate, %	Male	-8.18	0.33	(-8.82, -7.53)	3174	1.000
self-cure rate	Shared	0.21	0.06	(0.10, 0.30)	2539	1.000

Table 9: Posterior distributions of parameters, Model C

Variable	Sex	Mean	SD	95% CrI	ESS	$\hat{R}$
rate to symptomatic onset	Female	0.58	0.11	(0.41, 0.84)	2377	1.000
rate to case-detection, smear-	Female	0.14	0.02	(0.10, 0.20)	1340	1.000
rate to case-detection, smear+	Female	0.49	0.10	(0.33, 0.73)	2348	1.000
annual decline rate, %	Female	-7.43	0.43	(-8.28, -6.61)	3600	1.000
rate to symptomatic onset	Male	0.57	0.09	(0.41, 0.77)	1933	1.001
rate to case-detection, smear-	Male	0.10	0.02	(0.08, 0.14)	1663	1.001
rate to case-detection, smear+	Male	0.28	0.04	(0.21, 0.36)	2654	1.001
annual decline rate, %	Male	-8.12	0.34	(-8.77, -7.48)	2661	1.002
smear conversion rate	Shared	1.02	0.48	(0.10, 1.89)	781	1.002
smear+ at symptom onset	Shared	0.32	0.21	(0.02, 0.74)	953	1.000
rate to self-cure	Shared	0.21	0.06	(0.11, 0.30)	1820	1.002

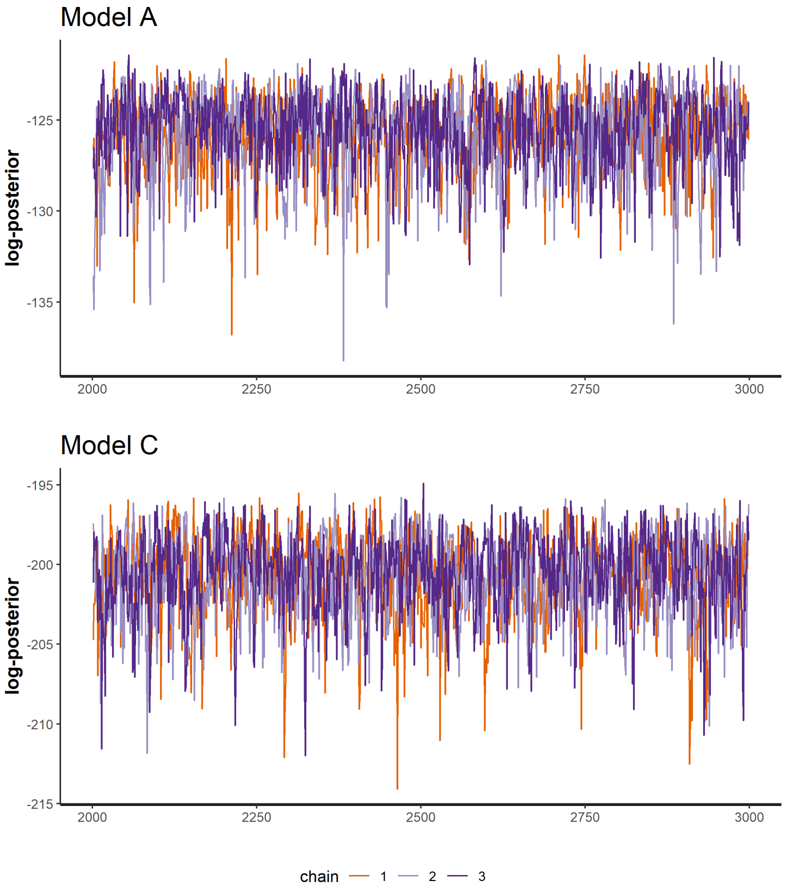

Figure 15: Trace of log-posterior probabilities

B.6 Malawi (MWI)

B.6.1 Data and model estimates

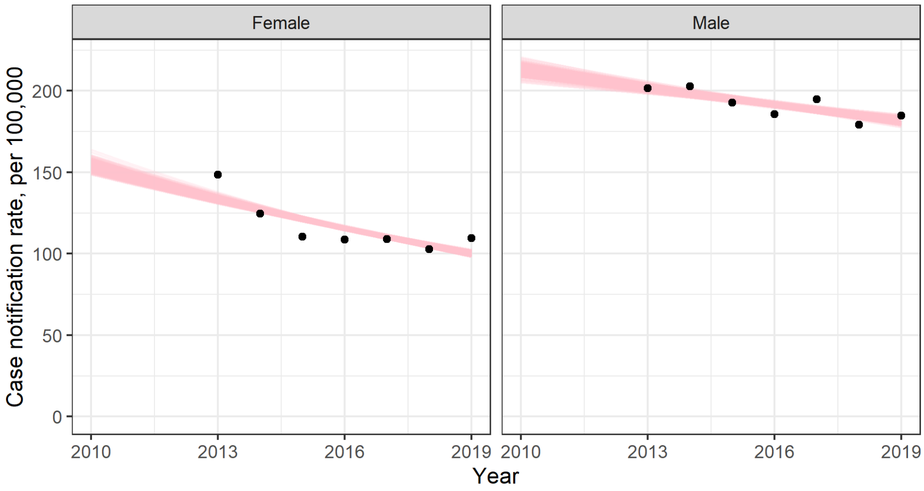

Figure 16: Case notification rate in model estimates and data by sex

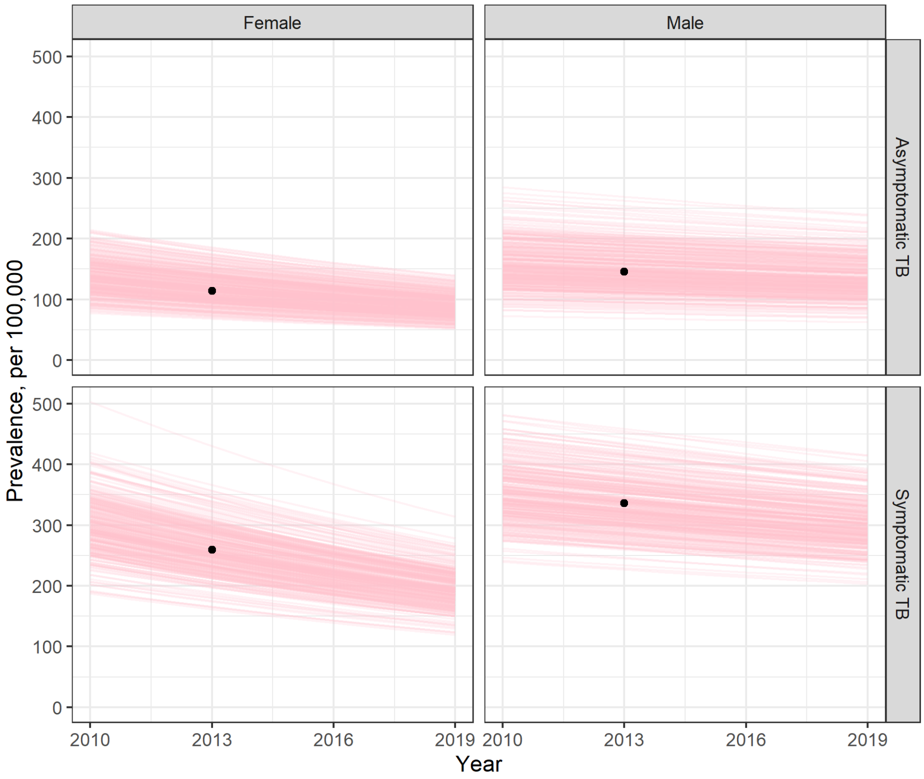

Figure 17: Prevalence in model estimates and data by sex and symptomatic or not

B.6.2 Posterior parameters

Table 10: Posterior distributions of parameters, Model B

Variable	Sex	Mean	SD	95% CrI	ESS	$\hat{R}$
rate to symptomatic onset	Female	1.75	0.43	(1.10, 2.76)	2648	0.999
rate to initial care-seeking	Female	1.43	0.33	(0.91, 2.20)	3180	1.001
rate to case-detection	Female	0.93	0.18	(0.64, 1.34)	2761	1.000
annual decline rate, %	Female	4.78	0.24	(4.32, 5.26)	3408	1.000
rate to symptomatic onset	Male	2.03	0.51	(1.26, 3.18)	2877	1.000
rate to initial care-seeking	Male	1.76	0.42	(1.11, 2.76)	3211	1.000
rate to case-detection	Male	1.14	0.24	(0.76, 1.66)	2845	0.999
annual decline rate, %	Male	1.76	0.21	(1.36, 2.17)	3892	1.000
self-cure rate	Shared	0.20	0.06	(0.10, 0.29)	3226	0.999

Table 11: Posterior distributions of parameters, Model C

Variable	Sex	Mean	SD	95% CrI	ESS	$\hat{R}$
rate to symptomatic onset	Female	1.69	0.39	(1.08, 2.59)	2717	1.002
rate to case-detection, smear-	Female	0.51	0.08	(0.37, 0.70)	2028	1.001
rate to case-detection, smear+	Female	0.63	0.13	(0.43, 0.94)	2395	1.000
annual decline rate, %	Female	4.80	0.25	(4.33, 5.29)	3411	1.000
rate to symptomatic onset	Male	2.02	0.50	(1.26, 3.20)	2198	1.000
rate to case-detection, smear-	Male	0.54	0.09	(0.40, 0.75)	1402	1.003
rate to case-detection, smear+	Male	0.72	0.14	(0.49, 1.06)	2289	1.000
annual decline rate, %	Male	1.77	0.21	(1.37, 2.19)	3794	1.000
smear conversion rate	Shared	0.35	0.25	(0.01, 0.86)	1576	1.003
smear+ at symptom onset	Shared	0.30	0.16	(0.01, 0.53)	1501	1.004
rate to self-cure	Shared	0.20	0.06	(0.11, 0.29)	3068	0.999

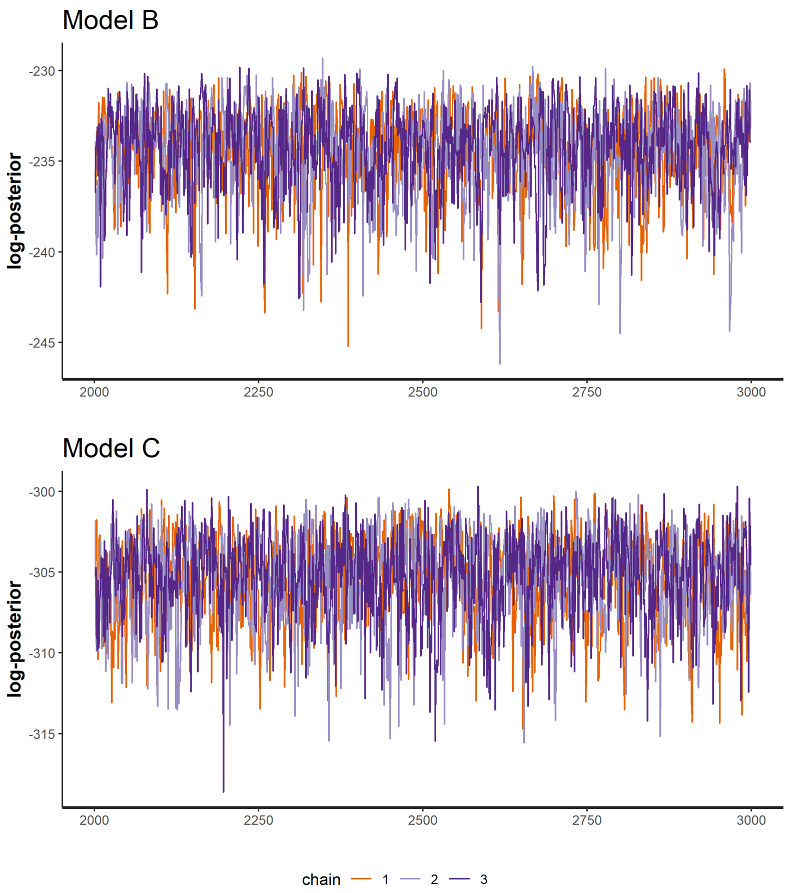

Figure 18: Trace of log-posterior probabilities

B.7 Pakistan (PAK)

B.7.1 Data and model estimates

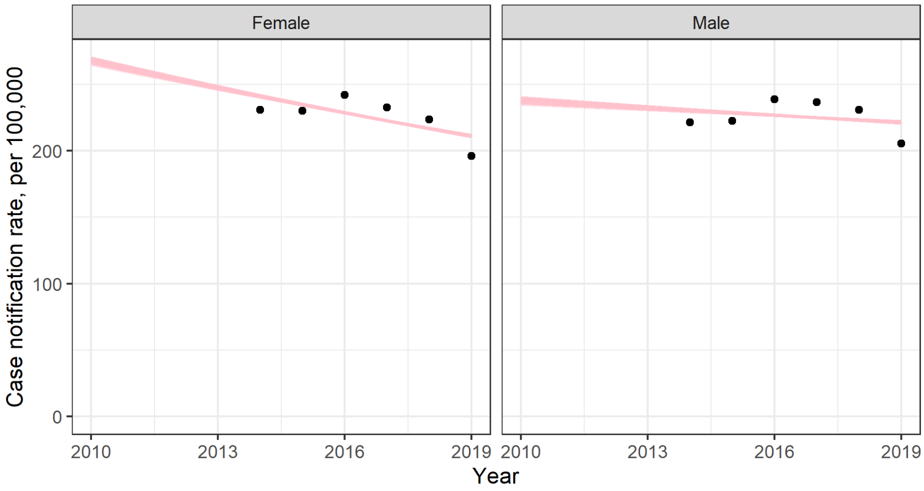

Figure 19: Case notification rate in model estimates and data by sex

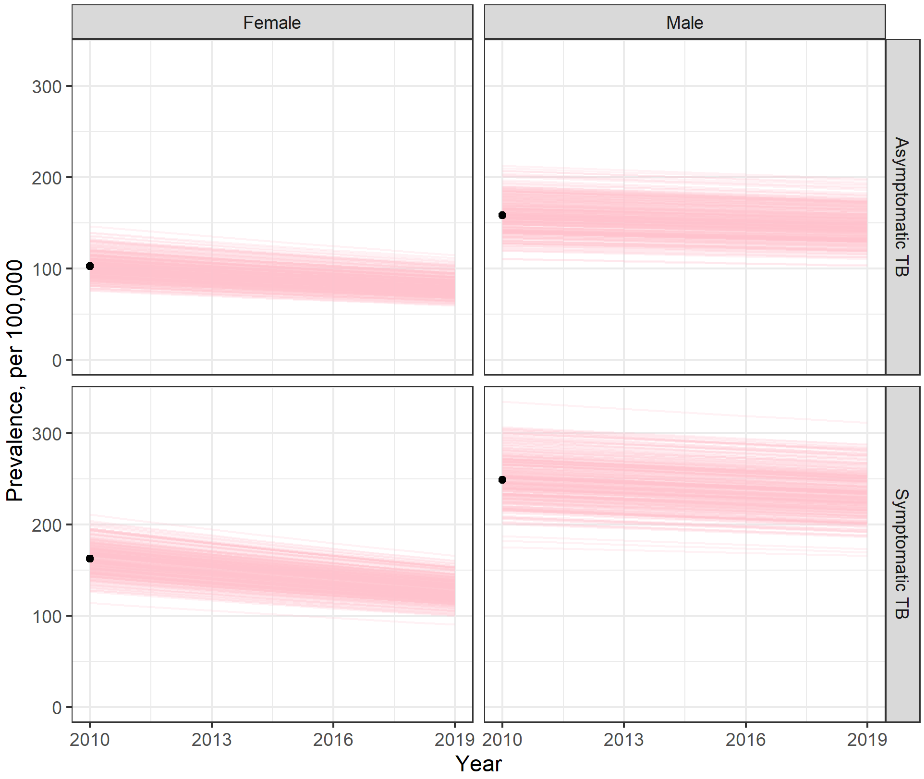

Figure 20: Prevalence in model estimates and data by sex and symptomatic or not

B.7.2 Posterior parameters

Table 12: Posterior distributions of parameters, Model A

Variable	Sex	Mean	SD	95% CrI	ESS	$\hat{R}$
rate to symptomatic onset	Female	3.01	0.40	(2.34, 3.93)	2963	0.999
rate to case-detection	Female	1.65	0.17	(1.36, 2.01)	2465	1.000
annual decline rate, %	Female	2.65	0.06	(2.53, 2.77)	4434	1.000
rate to symptomatic onset	Male	1.94	0.25	(1.51, 2.50)	3220	1.000
rate to case-detection	Male	0.96	0.09	(0.79, 1.16)	2322	1.000
annual decline rate, %	Male	0.80	0.06	(0.67, 0.92)	4191	1.000
self-cure rate	Shared	0.20	0.06	(0.10, 0.30)	3550	0.999

Table 13: Posterior distributions of parameters, Model C

Variable	Sex	Mean	SD	95% CrI	ESS	$\hat{R}$
rate to symptomatic onset	Female	2.98	0.38	(2.31, 3.81)	839	1.001
rate to case-detection, smear-	Female	2.53	0.38	(1.93, 3.41)	724	1.001
rate to case-detection, smear+	Female	1.33	0.17	(1.05, 1.72)	773	1.009
annual decline rate, %	Female	2.65	0.06	(2.53, 2.77)	1249	1.002
rate to symptomatic onset	Male	1.96	0.24	(1.53, 2.48)	1237	1.000
rate to case-detection, smear-	Male	1.87	0.24	(1.44, 2.38)	639	1.003
rate to case-detection, smear+	Male	0.63	0.07	(0.52, 0.78)	932	1.001
annual decline rate, %	Male	0.80	0.06	(0.67, 0.91)	1224	1.003
smear conversion rate	Shared	1.36	0.43	(0.47, 1.96)	482	1.003
smear+ at symptom onset	Shared	0.29	0.09	(0.13, 0.46)	464	1.001
rate to self-cure	Shared	0.18	0.06	(0.10, 0.29)	1315	1.002

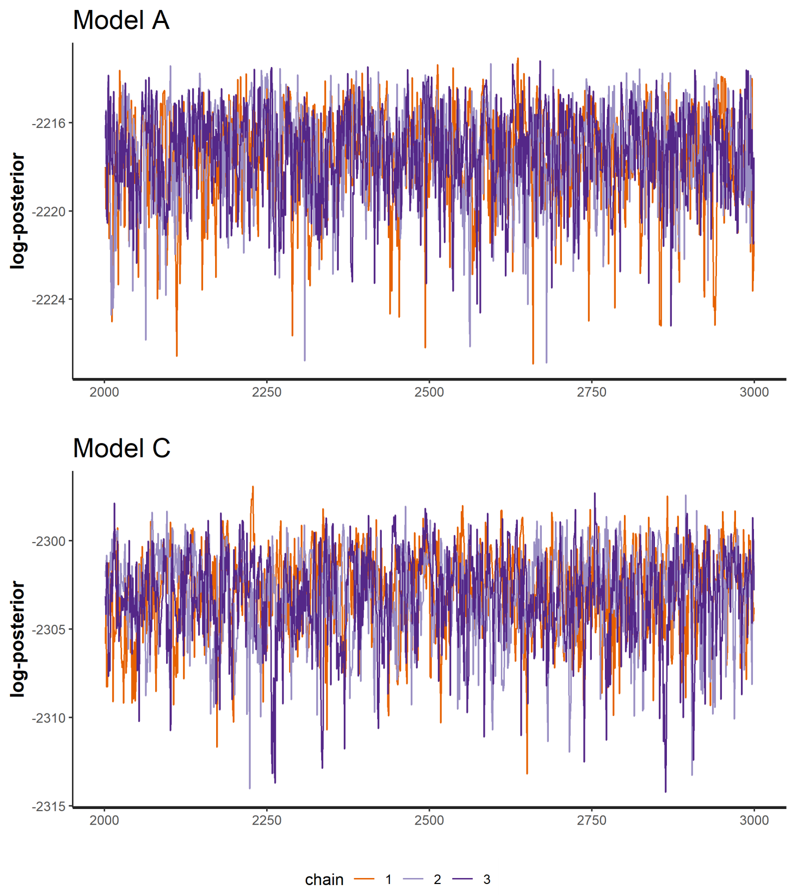

Figure 21: Trace of log-posterior probabilities

B.8 Philippines (PHL)

B.8.1 Data and model estimates

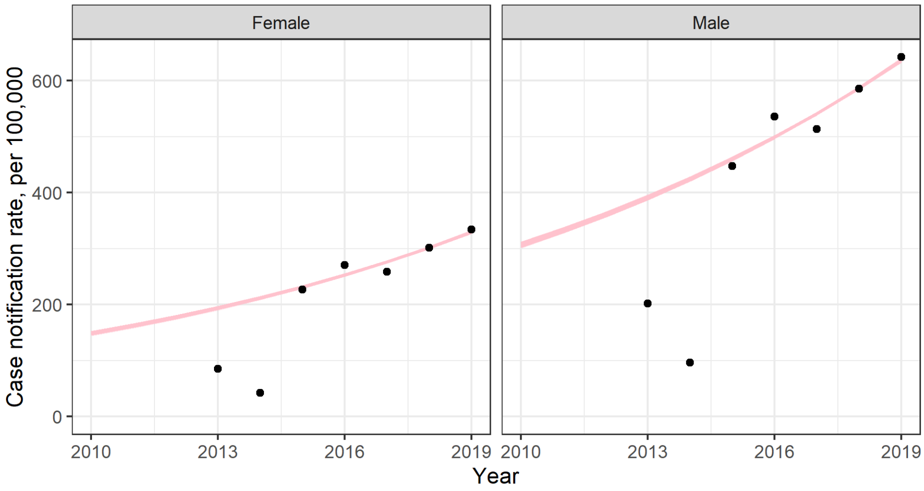

Figure 22: Case notification rate in model estimates and data by sex

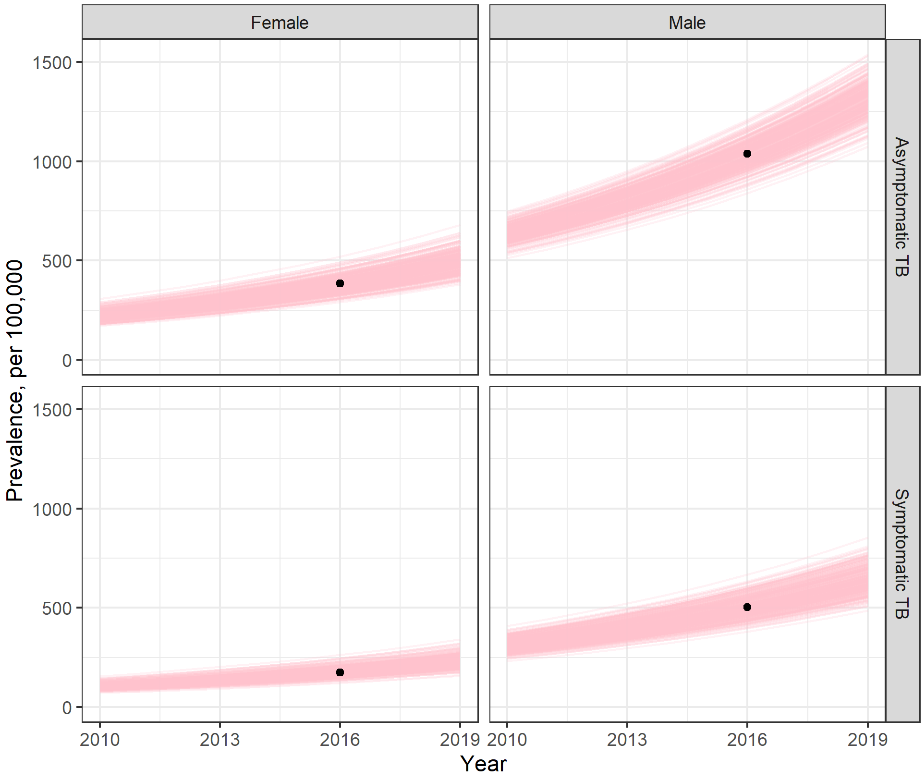

Figure 23: Prevalence in model estimates and data by sex and symptomatic or not

B.8.2 Posterior parameters

Table 14: Posterior distributions of parameters, Model B

Variable	Sex	Mean	SD	95% CrI	ESS	$\hat{R}$
rate to symptomatic onset	Female	0.84	0.09	(0.67, 1.04)	2846	1.000
rate to initial care-seeking	Female	3.39	0.71	(2.27, 5.08)	3061	1.000
rate to case-detection	Female	2.85	0.60	(1.90, 4.26)	3491	0.999
annual decline rate, %	Female	-8.83	0.10	(-9.03, -8.63)	3711	1.001
rate to symptomatic onset	Male	0.66	0.06	(0.56, 0.78)	3220	0.999
rate to initial care-seeking	Male	2.39	0.34	(1.80, 3.14)	3813	0.999
rate to case-detection	Male	1.97	0.27	(1.51, 2.55)	3331	1.000
annual decline rate, %	Male	-8.10	0.07	(-8.24, -7.97)	3910	0.999
self-cure rate	Shared	0.20	0.06	(0.10, 0.29)	3477	0.999

Table 15: Posterior distributions of parameters, Model C

Variable	Sex	Mean	SD	95% CrI	ESS	$\hat{R}$
rate to symptomatic onset	Female	0.84	0.09	(0.68, 1.02)	569	1.007
rate to case-detection, smear-	Female	1.83	0.31	(1.34, 2.58)	477	1.002
rate to case-detection, smear+	Female	1.24	0.25	(0.85, 1.84)	676	1.001
annual decline rate, %	Female	-8.83	0.10	(-9.03, -8.64)	1017	1.001
rate to symptomatic onset	Male	0.67	0.06	(0.57, 0.78)	530	1.001
rate to case-detection, smear-	Male	1.40	0.19	(1.06, 1.82)	596	1.001
rate to case-detection, smear+	Male	0.74	0.09	(0.58, 0.94)	693	1.002
annual decline rate, %	Male	-8.10	0.07	(-8.24, -7.97)	1006	1.002
smear conversion rate	Shared	0.88	0.52	(0.05, 1.85)	438	1.004
smear+ at symptom onset	Shared	0.26	0.14	(0.02, 0.49)	421	1.003
rate to self-cure	Shared	0.20	0.06	(0.11, 0.30)	884	1.003

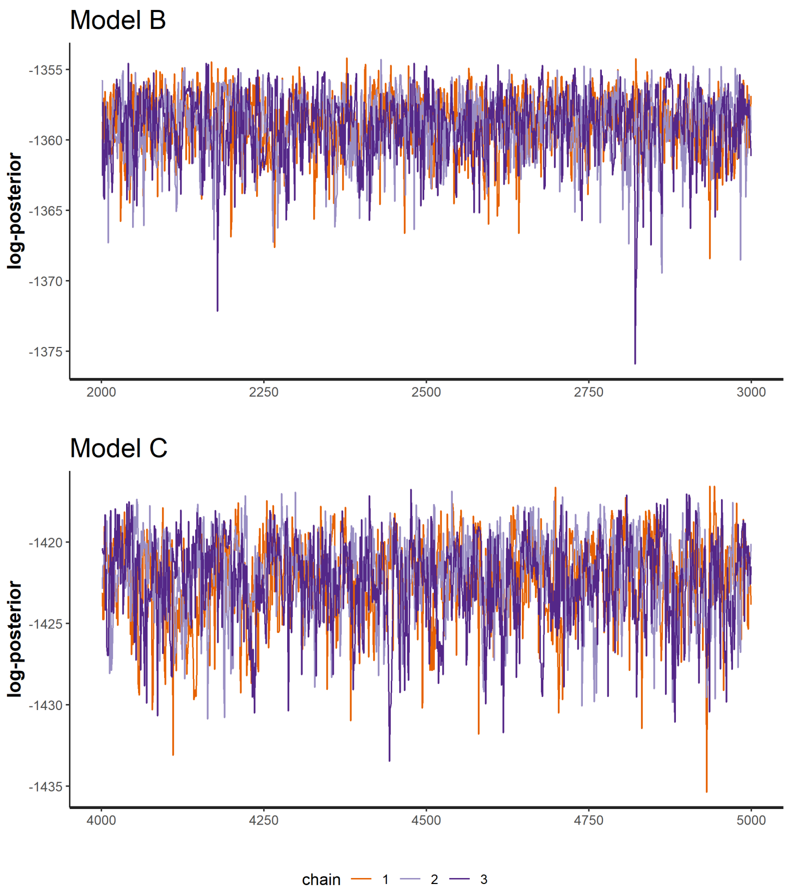

Figure 24: Trace of log-posterior probabilities

B.9 Uganda (UGA)

B.9.1 Data and model estimates

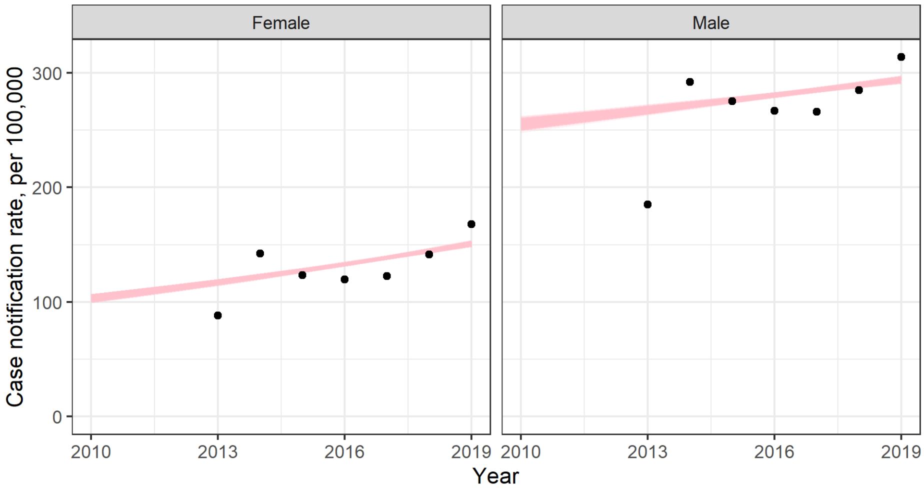

Figure 25: Case notification rate in model estimates and data by sex

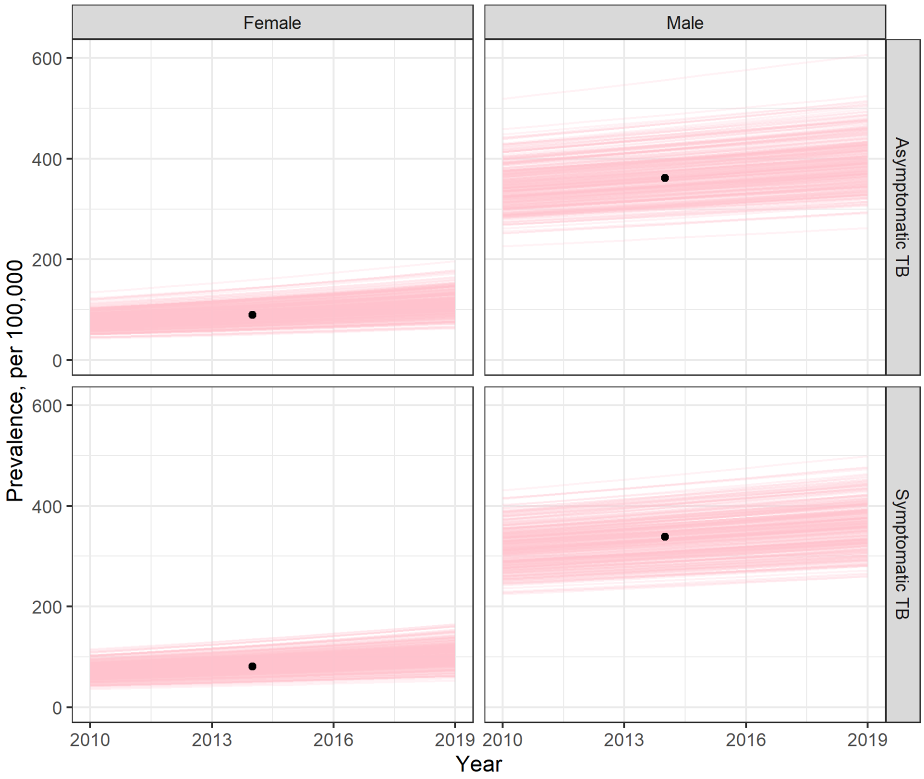

Figure 26: Prevalence in model estimates and data by sex and symptomatic or not

B.9.2 Posterior parameters

Table 16: Posterior distributions of parameters, Model B

Variable	Sex	Mean	SD	95% CrI	ESS	$\hat{R}$
rate to symptomatic onset	Female	1.69	0.38	(1.12, 2.58)	2384	1.000
rate to initial care-seeking	Female	5.59	2.50	(2.54, 12.06)	2274	1.000
rate to case-detection	Female	2.26	0.66	(1.29, 3.84)	2470	1.000
annual decline rate, %	Female	-4.22	0.19	(-4.61, -3.85)	3207	1.000
rate to symptomatic onset	Male	1.06	0.16	(0.80, 1.42)	2497	0.999
rate to initial care-seeking	Male	2.12	0.43	(1.44, 3.09)	2711	0.999
rate to case-detection	Male	1.53	0.27	(1.09, 2.14)	2513	0.999
annual decline rate, %	Male	-1.58	0.14	(-1.86, -1.31)	3819	1.000
self-cure rate	Shared	0.20	0.06	(0.10, 0.29)	3035	1.000

Table 17: Posterior distributions of parameters, Model C

Variable	Sex	Mean	SD	95% CrI	ESS	$\hat{R}$
rate to symptomatic onset	Female	1.69	0.39	(1.09, 2.56)	2491	1.001
rate to case-detection, smear-	Female	0.69	0.14	(0.49, 1.03)	916	1.002
rate to case-detection, smear+	Female	2.68	0.96	(1.40, 5.11)	2241	1.000
annual decline rate, %	Female	-4.22	0.20	(-4.61, -3.83)	3199	1.000
rate to symptomatic onset	Male	1.03	0.15	(0.78, 1.35)	2488	1.001
rate to case-detection, smear-	Male	0.55	0.09	(0.40, 0.74)	1431	0.999
rate to case-detection, smear+	Male	1.16	0.22	(0.81, 1.68)	2273	1.001
annual decline rate, %	Male	-1.58	0.14	(-1.84, -1.31)	3715	1.000
smear conversion rate	Shared	0.72	0.51	(0.02, 1.72)	1070	1.001
smear+ at symptom onset	Shared	0.36	0.20	(0.02, 0.65)	1059	1.001
rate to self-cure	Shared	0.20	0.06	(0.10, 0.29)	3084	1.000

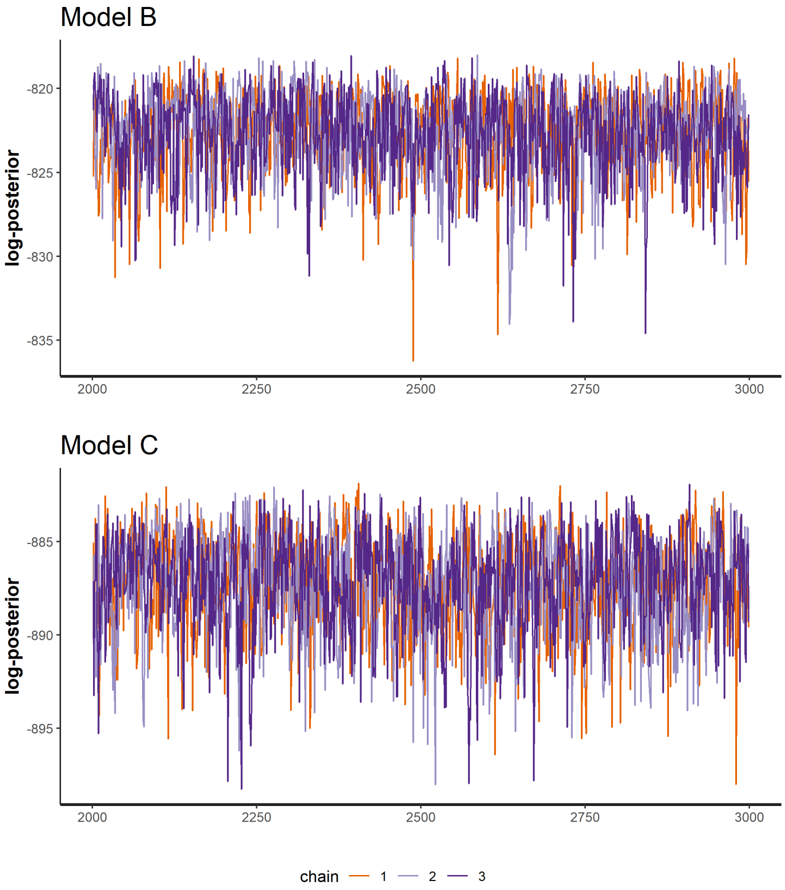

Figure 27: Trace of log-posterior probabilities

B.10 Tanzania (TZA)

B.10.1 Data and model estimates

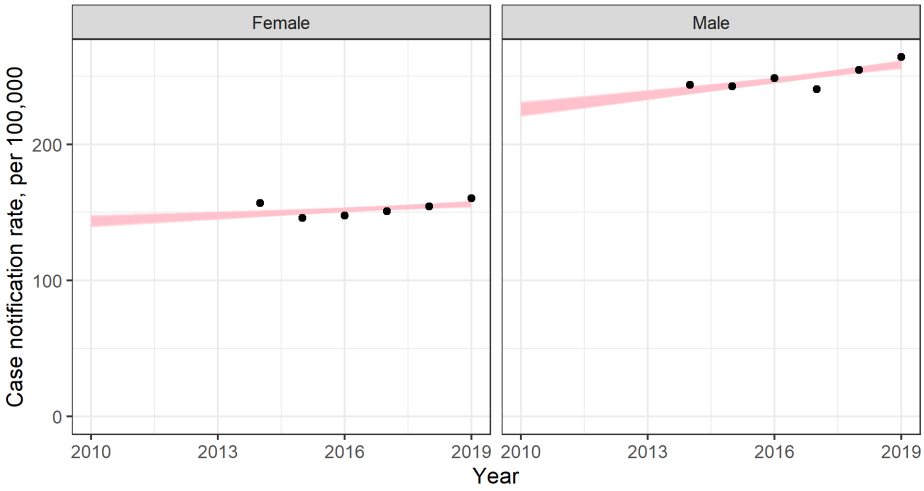

Figure 28: Case notification rate in model estimates and data by sex

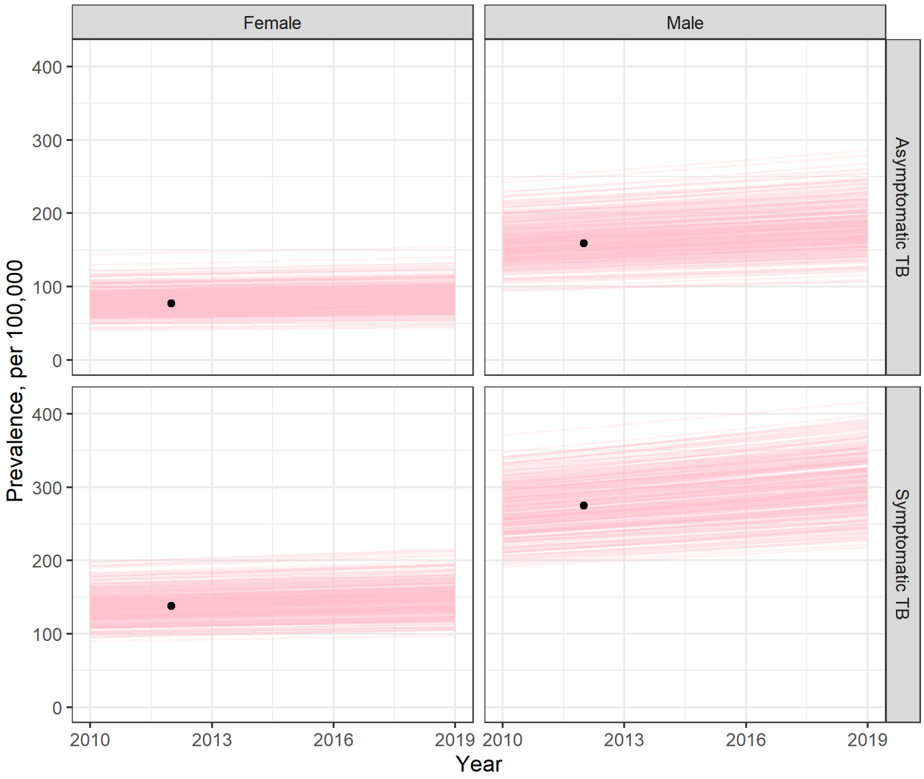

Figure 29: Prevalence in model estimates and data by sex and symptomatic or not

B.10.2 Posterior parameters

Table 18: Posterior distributions of parameters, Model B

Variable	Sex	Mean	SD	95% CrI	ESS	$\hat{R}$
rate to symptomatic onset	Female	2.44	0.55	(1.59, 3.77)	2076	1.001
rate to initial care-seeking	Female	1.94	0.40	(1.32, 2.87)	1983	1.002
rate to case-detection	Female	2.76	0.69	(1.75, 4.39)	2065	1.000
annual decline rate, %	Female	-0.94	0.16	(-1.25, -0.63)	3047	0.999
rate to symptomatic onset	Male	2.02	0.38	(1.42, 2.88)	2506	0.999
rate to initial care-seeking	Male	1.55	0.27	(1.11, 2.17)	2158	1.001
rate to case-detection	Male	2.22	0.48	(1.50, 3.32)	2276	1.000
annual decline rate, %	Male	-1.52	0.12	(-1.77, -1.29)	3650	0.999
self-cure rate	Shared	0.20	0.06	(0.10, 0.30)	3052	1.000

Table 19: Posterior distributions of parameters, Model C

Variable	Sex	Mean	SD	95% CrI	ESS	$\hat{R}$
rate to symptomatic onset	Female	2.45	0.53	(1.64, 3.66)	892	1.000
rate to case-detection, smear-	Female	2.92	0.80	(1.67, 4.82)	714	1.004
rate to case-detection, smear+	Female	0.72	0.13	(0.51, 1.00)	519	1.011
annual decline rate, %	Female	-0.94	0.16	(-1.24, -0.63)	1113	1.001
rate to symptomatic onset	Male	2.04	0.38	(1.45, 2.90)	763	1.002
rate to case-detection, smear-	Male	2.41	0.62	(1.45, 3.83)	676	1.003
rate to case-detection, smear+	Male	0.57	0.08	(0.42, 0.74)	699	1.006
annual decline rate, %	Male	-1.52	0.12	(-1.76, -1.28)	968	1.001
smear conversion rate	Shared	1.07	0.56	(0.05, 1.93)	402	1.006
smear+ at symptom onset	Shared	0.48	0.09	(0.29, 0.62)	233	1.010
rate to self-cure	Shared	0.19	0.06	(0.10, 0.29)	973	1.000

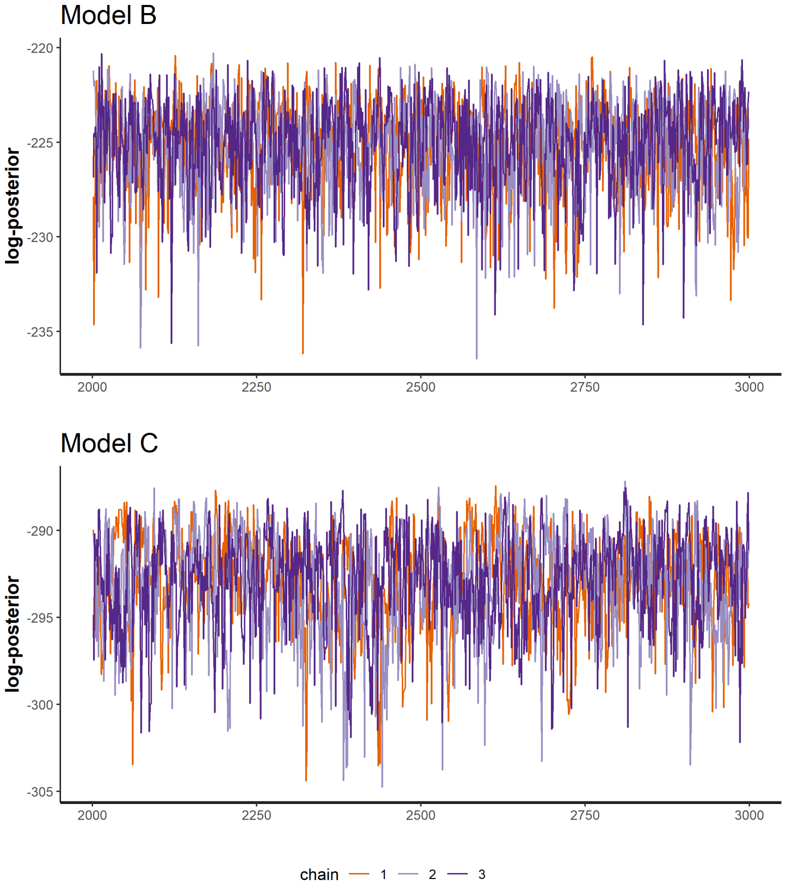

Figure 30: Trace of log-posterior probabilities

B.11 Vietnam (VNM)

B.11.1 Data and model estimates

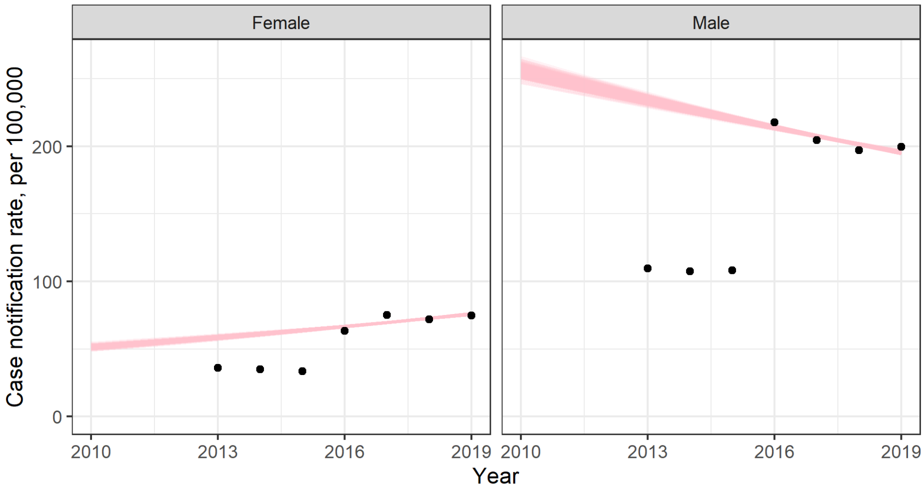

Figure 31: Case notification rate in model estimates and data by sex

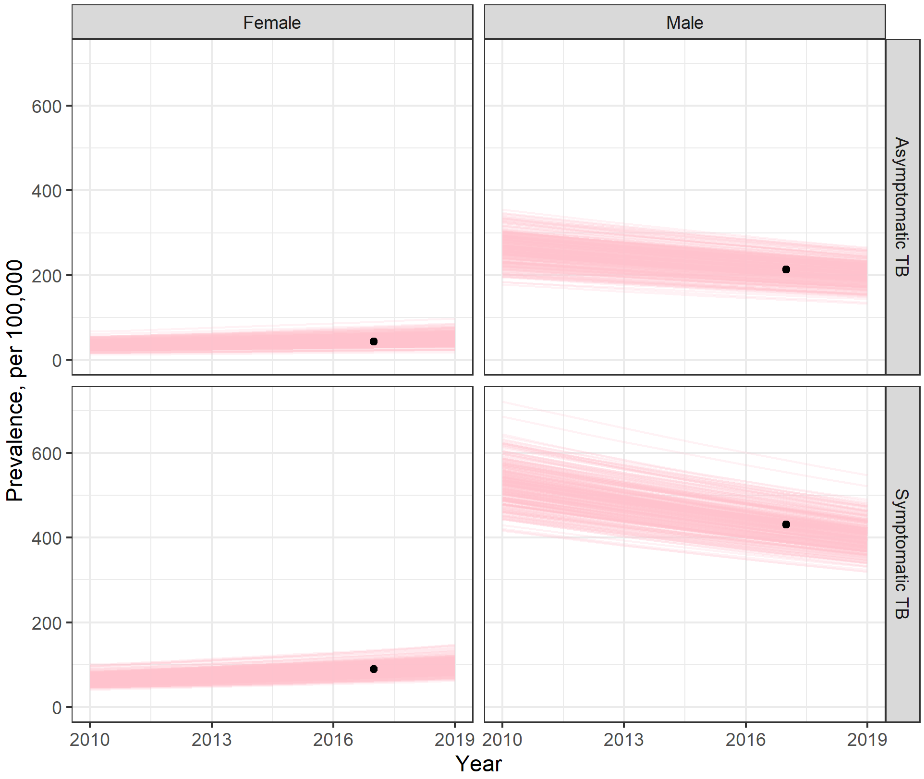

Figure 32: Prevalence in model estimates and data by sex and symptomatic or not

B.11.2 Posterior parameters

Table 20: Posterior distributions of parameters, Model A

Variable	Sex	Mean	SD	95% CrI	ESS	$\hat{R}$
rate to symptomatic onset	Female	2.34	0.64	(1.39, 3.87)	2333	1.001
rate to case-detection	Female	0.79	0.13	(0.57, 1.09)	1586	1.002
annual decline rate, %	Female	-4.32	0.27	(-4.85, -3.77)	3549	1.000
rate to symptomatic onset	Male	1.53	0.24	(1.12, 2.06)	2556	1.000
rate to case-detection	Male	0.49	0.05	(0.40, 0.58)	1377	1.000
annual decline rate, %	Male	3.02	0.16	(2.68, 3.34)	3750	0.999
self-cure rate	Shared	0.20	0.06	(0.10, 0.29)	2778	1.000

Table 21: Posterior distributions of parameters, Model C

Variable	Sex	Mean	SD	95% CrI	ESS	$\hat{R}$
rate to symptomatic onset	Female	2.31	0.64	(1.39, 3.83)	2020	1.002
rate to case-detection, smear-	Female	0.27	0.03	(0.20, 0.34)	953	1.003
rate to case-detection, smear+	Female	2.06	0.60	(1.18, 3.51)	1896	1.000
annual decline rate, %	Female	-4.32	0.27	(-4.85, -3.82)	3327	0.999
rate to symptomatic onset	Male	1.38	0.21	(1.02, 1.85)	1905	1.001
rate to case-detection, smear-	Male	0.21	0.02	(0.17, 0.26)	1178	1.002
rate to case-detection, smear+	Male	1.57	0.33	(1.07, 2.33)	2295	1.000
annual decline rate, %	Male	3.02	0.17	(2.69, 3.34)	3024	1.000
smear conversion rate	Shared	0.09	0.10	(0.00, 0.39)	92	1.038
smear+ at symptom onset	Shared	0.51	0.10	(0.22, 0.62)	118	1.035
rate to self-cure	Shared	0.18	0.06	(0.10, 0.29)	1804	1.001

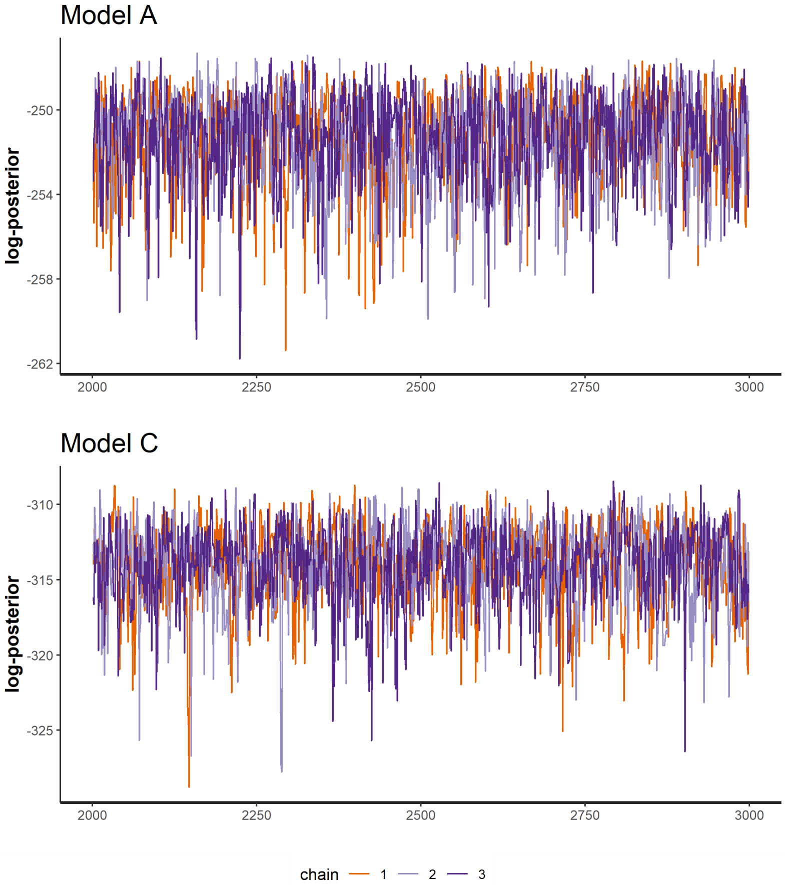

Figure 33: Trace of log-posterior probabilities

B.12 Zambia (ZMB)

B.12.1 Data and model estimates

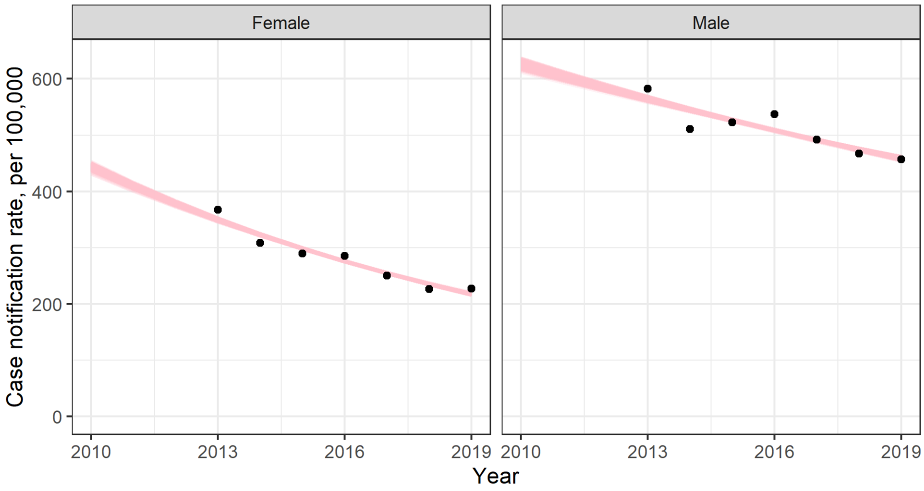

Figure 34: Case notification rate in model estimates and data by sex

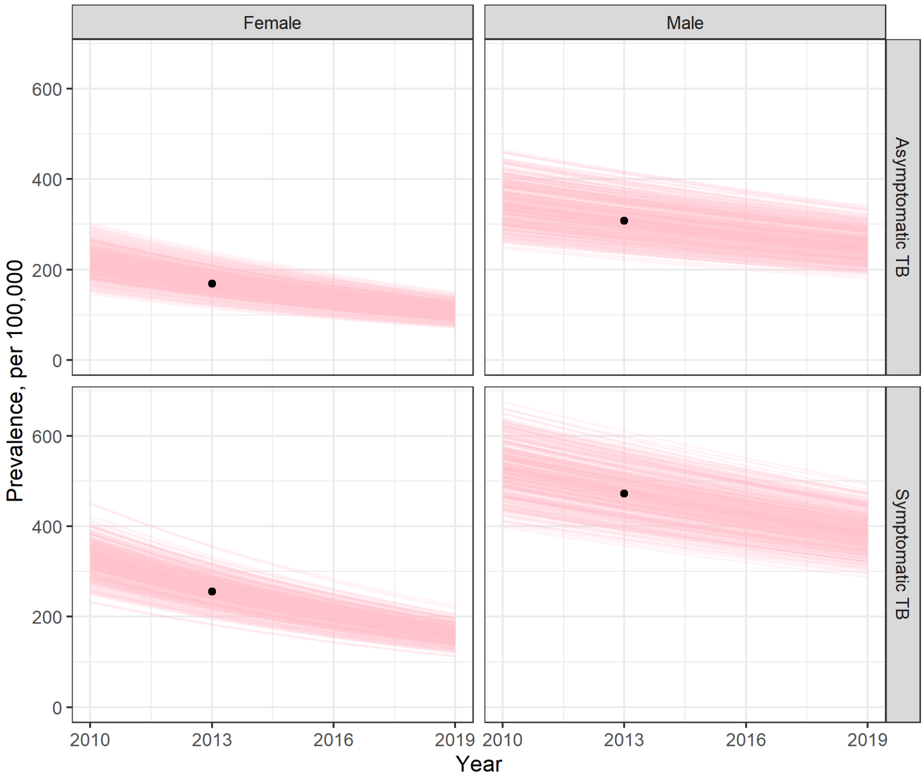

Figure 35: Prevalence in model estimates and data by sex and symptomatic or not

B.12.2 Posterior parameters

Table 22: Posterior distributions of parameters, Model B

Variable	Sex	Mean	SD	95% CrI	ESS	$\hat{R}$
rate to symptomatic onset	Female	2.37	0.35	(1.78, 3.14)	2588	1.002
rate to initial care-seeking	Female	2.96	0.51	(2.11, 4.13)	2716	1.000
rate to case-detection	Female	2.75	0.48	(1.96, 3.87)	2673	1.000
annual decline rate, %	Female	7.92	0.17	(7.57, 8.26)	3407	1.001
rate to symptomatic onset	Male	2.22	0.31	(1.68, 2.92)	2543	1.000
rate to initial care-seeking	Male	2.64	0.40	(1.96, 3.51)	2789	1.001
rate to case-detection	Male	2.40	0.36	(1.77, 3.19)	2497	1.000
annual decline rate, %	Male	3.46	0.13	(3.22, 3.70)	3295	1.000
self-cure rate	Shared	0.20	0.06	(0.10, 0.30)	2998	0.999

Table 23: Posterior distributions of parameters, Model C

Variable	Sex	Mean	SD	95% CrI	ESS	$\hat{R}$
rate to symptomatic onset	Female	2.37	0.37	(1.75, 3.19)	3094	1.000
rate to case-detection, smear-	Female	1.62	0.20	(1.26, 2.07)	1894	1.001
rate to case-detection, smear+	Female	1.22	0.20	(0.89, 1.66)	2139	1.003
annual decline rate, %	Female	7.92	0.17	(7.58, 8.26)	4731	0.999
rate to symptomatic onset	Male	2.22	0.32	(1.68, 2.90)	3091	1.000
rate to case-detection, smear-	Male	1.44	0.17	(1.13, 1.80)	1713	1.001
rate to case-detection, smear+	Male	0.98	0.14	(0.75, 1.28)	2352	1.001
annual decline rate, %	Male	3.45	0.12	(3.22, 3.70)	4550	1.000
smear conversion rate	Shared	0.66	0.43	(0.03, 1.47)	1180	1.002
smear+ at symptom onset	Shared	0.24	0.13	(0.01, 0.44)	1204	1.002
rate to self-cure	Shared	0.20	0.06	(0.10, 0.29)	3540	0.999

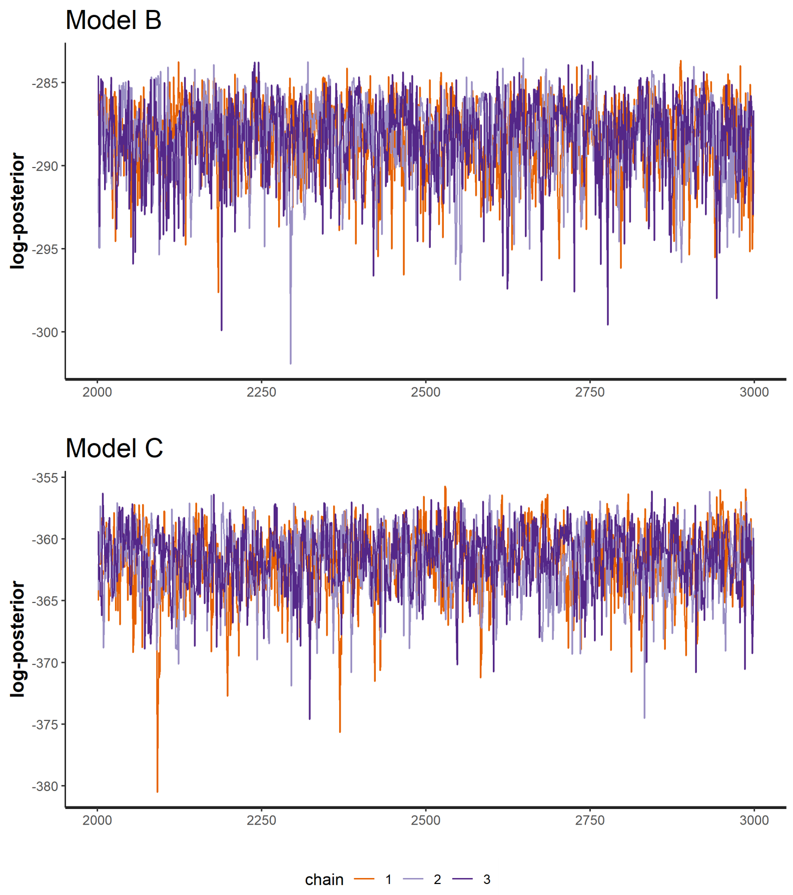

Figure 36: Trace of log-posterior probabilities

C Inference: epidemiological indices

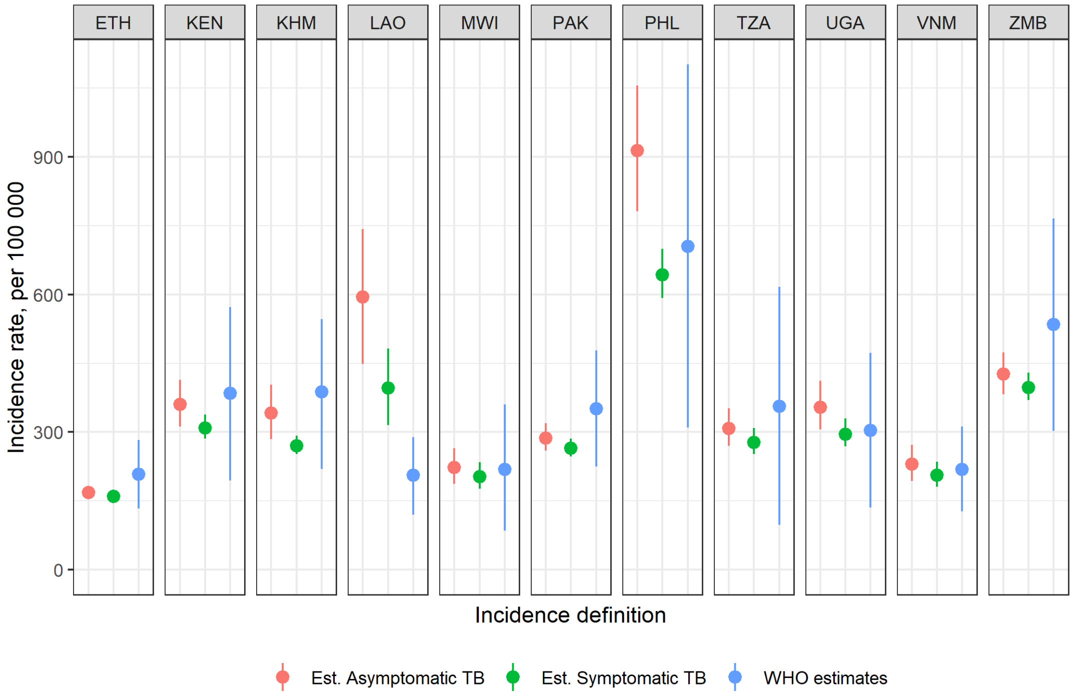

Figure 37: **Incidence estimates considering the asymptomatic period in 2019.** The country labels: KHM=Cambodia, ETH=Ethiopia, KEN=Kenya, LAO=Lao People's Democratic Republic, MWI=Malawi, PAK=Pakistan, PHL= Philippines, TZA=United Republic of Tanzania, UGA=Uganda, VNM=Viet Nam, ZMB=Zambia

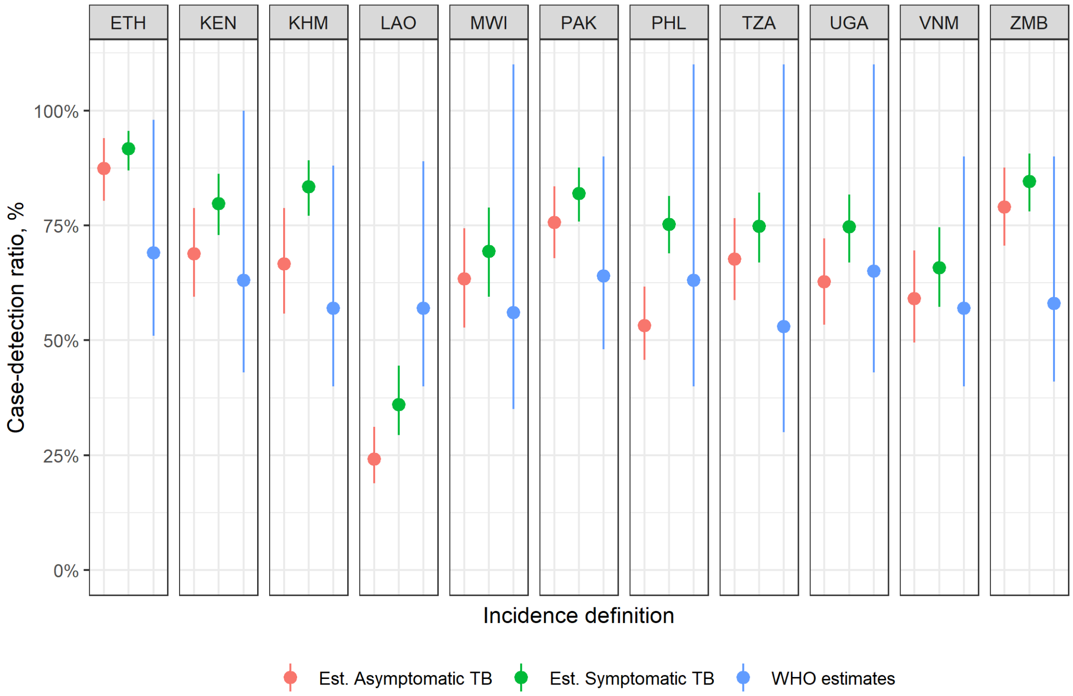

Figure 38: **Case detection ratio estimates considering the asymptomatic period.** Country labels: KHM=Cambodia, ETH=Ethiopia, KEN=Kenya, LAO=Lao People's Democratic Republic, MWI=Malawi, PAK=Pakistan, PHL= Philippines, TZA=United Republic of Tanzania, UGA=Uganda, VNM=Viet Nam, ZMB=Zambia

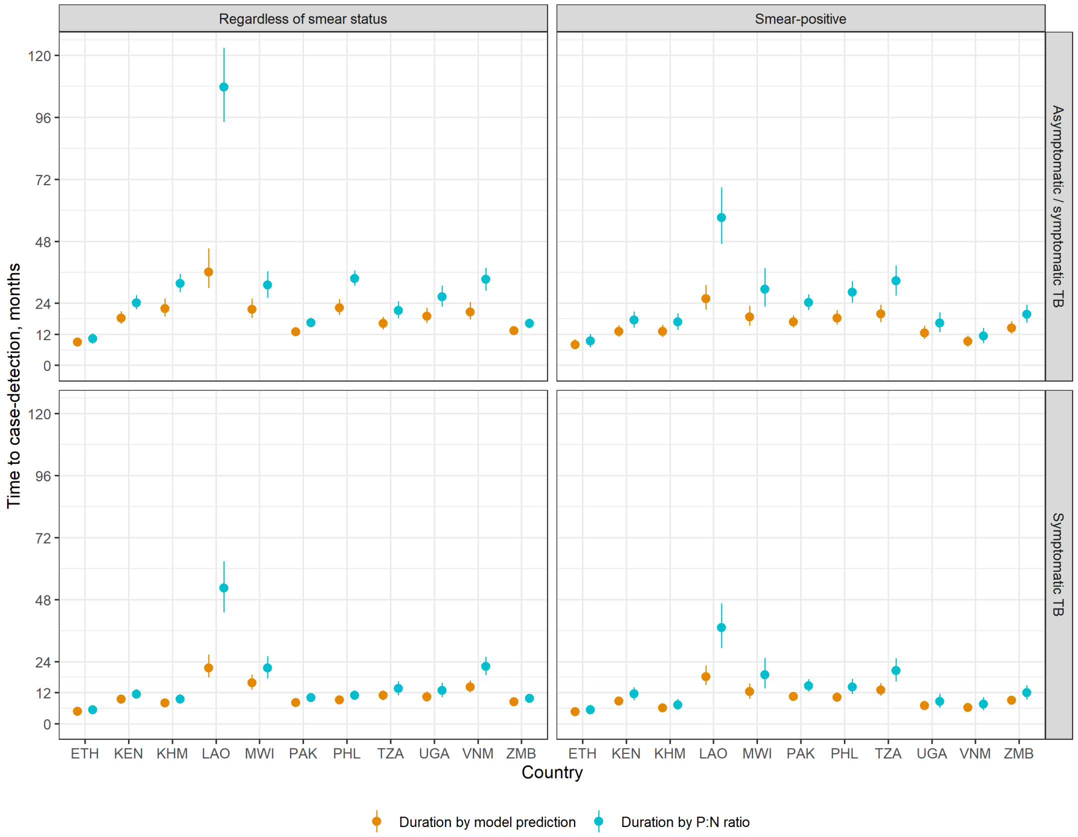

Figure 39: **Delay to case-detection estimates by prevalence-to-notification ratio and model prediction.** The upper sub-figure evaluates the time to case-detection since TB-detectable (D1), and the lower evaluates the time to case-detection since first symptom developed (D2). The evaluations are based on 2019 estimators, and the prevalence used the model forecasts for 2019. P:N ratio for D1 used the prevalence defined on bacterially confirmed TB and P:N ratio for D2 used the symptomatic prevalent TB only. The durations by model prediction considers the competing events of self-cure and deaths before case-detection. Country labels: KHM=Cambodia, ETH=Ethiopia, KEN=Kenya, LAO=Lao People's Democratic Republic, MWI=Malawi, PAK=Pakistan, PHL= Philippines, TZA=United Republic of Tanzania, UGA=Uganda, VNM=Viet Nam, ZMB=Zambia

D Inference: delay and healthcare cascade

D.1 Estimation of delay and duration

The duration of a state s is defined as the expected time to any transition as

$$Duration_s \equiv E[min(T_d, T_o)]$$

Where T_d denotes the time to case-detection and T_o denotes the time to any other event; T_d and T_o are independent non-negative continuous random variables. On the other hand, the delay of a state s to case-detection is defined as the expected time to case-detection conditioned on case-detection being sooner than the other events.

$$Delay_s \equiv E[T_d|T_d < T_o] = E[min(T_d, T_o)|T_d < T_o]$$

As a result, $Duration_s = Delay_s$ when $min(T_d, T_o)$ and $T_d < T_o$ are independent, for example, T_d and T_o are exponentially distributed (see the end of this chapter for the proof).

Assuming T_d and T_o are exponentially distributed with rates of r_d and r_o, respectively, $min(T_d, T_o)$ is an Exponential random variable with rate of $r_d + r_o$. Thus,

$$Delay_s = Duration_s = \frac{1}{r_d + r_o}$$

Case-detection delay and prevalence-to-notification ratio For Model 1 with Symptomatic TB S and Asymptomatic TB A. We compare the three estimators for the delay to case-detection.

- Prevalence-to-notification ratio with all active TB (A, S), conventional approach:

$$D_1 = \frac{Prevalence_A + Prevalence_S}{Notification}$$

- Prevalence-to-notification ratio with all active TB (S):

$$D_2 = \frac{Prevalence_S}{Notification} = \frac{1}{r_d}$$

- Duration derived from this appendix:

$$D_3 = \frac{1}{r_d + r_o}$$

Firstly,

$$D_1 = \frac{Prevalence_A + Prevalence_S}{Notification} > \frac{Prevalence_S}{Notification} = D_2$$

Secondly,

$$D_2 = \frac{1}{r_d} > \frac{1}{r_d + r_o} = D_3$$

So

$$D_1 > D_2 > D_3$$

Lemma 1. *Given two exponentially distributed random variables T_d and T_o with rates of r_d and r_o respectively. $min(T_d, T_o)$ and $T_d < T_o$ are independent.*

Proof.

$$\begin{aligned} P[T_d < T_o | min(T_d, T_o) = t] &= \frac{P[T_d < T_o, min(T_d, T_o) = t]}{P[min(T_d, T_o) = t]} \\ &= \frac{P[T_d = t, T_o > t]}{P[T_d = t, T_o > t] + P[T_o = t, T_d > t]} \qquad independence\, of\, T_o\, and\, T_d \\ &= \frac{f_d(t)(1 - F_o(t))}{f_d(t)(1 - F_o(t)) + f_o(t)(1 - F_d(t))} \\ &= \frac{f_d(t)/(1 - F_d(t))}{f_d(t)/(1 - F_d(t)) + f_o(t)/(1 - F_o(t))} \\ &= \frac{h_d(t)}{h_d(t) + h_o(t)} \end{aligned}$$

Where $f(.)$, and $F(.)$ denote probability density function and cumulative density function;$h_d(t)$ and $h_o(t)$ denote the hazard functions of case detection and the other events respectively. For the Exponential distributions, the hazard functions: $h_d(t) = r_d$ and $h_o(t) = r_o$, which are constants regardless of time. Then,

$$\begin{aligned} P[T_d < T_o | min(T_d, T_o)] &= \frac{h_d(t)}{h_d(t) + h_o(t)} \\ &= \frac{r_d}{r_d + r_o} \\ &= P[T_d < T_o] \end{aligned}$$

That is, $T_d < T_o$ and $min(T_d, T_o)$ are independent. ■

E Inference: sensitivity analyses

E.1 Mortality

We conducted sensitivity analyses on the assumption of background mortality, excess mortality for asymptomatic TB and symptomatic TB across the countries of interest. For Blantyre and Kenya with data on HIV status, we tested the sensitivity analysis on the assumption of different level of untreated death of TB for people living with HIV.

Table 24: Sensitivity analysis: assumptions on untreated TB mortality rates

Scenario	M0	M1	M2	M3	M4
**Assumptions**					
Background mortality	0	μ			
Excess mortality for asymptomatic TB	0				50% / 10 years
Excess mortality for symptomatic TB	0		50% / 10 years	Average over smear status<br>Smear-: 50% / 10 years<br>Smear+: 70% / 10 years	
**Duration of asymptomatic TB**					
Cambodia	14.95 (12.22 – 18.52)	14.41 (11.89 – 17.69)	14.37 (11.83 – 17.58)	13.91 (11.51 – 16.96)	13.60 (11.23 – 16.49)
Ethiopia	4.40 (3.39 – 5.58)	4.40 (3.35 – 5.62)	4.36 (3.31 – 5.55)	4.28 (3.29 – 5.40)	4.24 (3.24 – 5.43)
Kenya	9.31 (7.61 – 11.33)	9.22 (7.53 – 11.33)	9.08 (7.42 – 11.08)	8.76 (7.10 – 10.73)	8.63 (7.11 – 10.51)
Lao PDR	17.13 (12.86 – 24.17)	16.22 (12.19 – 22.38)	15.98 (12.05 – 21.95)	14.52 (10.99 – 19.54)	14.11 (10.76 – 18.78)
Malawi	6.64 (4.67 – 9.18)	6.44 (4.59 – 8.86)	6.33 (4.45 – 8.72)	6.00 (4.12 – 8.24)	5.89 (4.14 – 8.10)
Pakistan	5.16 (4.18 – 6.25)	5.09 (4.16 – 6.14)	5.00 (4.09 – 6.11)	4.86 (3.97 – 5.86)	4.82 (3.94 – 5.80)
Philippines	13.99 (11.64 – 17.01)	13.65 (11.32 – 16.49)	13.44 (11.18 – 16.42)	13.13 (10.94 – 15.96)	12.89 (10.71 – 15.53)
Tanzania	5.73 (4.23 – 7.60)	5.59 (4.18 – 7.19)	5.52 (4.16 – 7.09)	5.29 (3.94 – 6.87)	5.23 (3.90 – 6.84)
Uganda	9.42 (7.24 – 11.95)	9.26 (7.14 – 11.68)	9.11 (7.17 – 11.43)	8.72 (6.83 – 11.03)	8.55 (6.66 – 10.75)
Vietnam	7.38 (5.47 – 9.68)	7.20 (5.34 – 9.52)	6.99 (5.29 – 9.18)	6.50 (4.98 – 8.36)	6.47 (4.89 – 8.35)
Zambia	5.23 (4.18 – 6.40)	5.18 (4.12 – 6.38)	5.10 (4.10 – 6.32)	4.98 (3.98 – 6.12)	4.93 (3.98 – 6.04)
**Duration of symptomatic TB**					
Cambodia	8.24 (6.68 – 9.98)	8.15 (6.61 – 9.78)	8.08 (6.68 – 9.69)	7.74 (6.38 – 9.27)	7.75 (6.39 – 9.24)
Ethiopia	4.92 (3.81 – 6.22)	4.93 (3.84 – 6.19)	4.88 (3.77 – 6.07)	4.78 (3.70 – 6.02)	4.78 (3.74 – 5.96)
Kenya	9.48 (8.03 – 11.15)	9.44 (7.95 – 11.14)	9.27 (7.83 – 10.91)	8.89 (7.63 – 10.30)	8.89 (7.58 – 10.39)
Lao PDR	27.69 (22.11 – 36.99)	26.46 (21.24 – 34.63)	25.42 (20.58 – 32.65)	21.60 (18.02 – 26.91)	21.56 (17.92 – 26.49)
Malawi	15.77 (12.80 – 19.43)	15.46 (12.49 – 18.92)	15.05 (12.21 – 18.43)	14.13 (11.72 – 17.01)	14.11 (11.54 – 17.05)
Pakistan	8.72 (7.55 – 10.02)	8.67 (7.56 – 9.97)	8.49 (7.43 – 9.75)	8.20 (7.15 – 9.40)	8.22 (7.20 – 9.41)
Philippines	9.23 (7.89 – 10.80)	9.13 (7.79 – 10.68)	8.99 (7.70 – 10.51)	8.69 (7.42 – 10.05)	8.67 (7.49 – 10.05)
Tanzania	10.87 (8.91 – 13.13)	10.70 (8.75 – 12.94)	10.51 (8.62 – 12.73)	10.03 (8.47 – 11.96)	10.06 (8.48 – 11.95)
Uganda	10.53 (8.48 – 12.89)	10.40 (8.33 – 12.70)	10.16 (8.30 – 12.35)	9.66 (7.98 – 11.61)	9.61 (7.92 – 11.47)
Vietnam	16.20 (13.52 – 19.43)	15.91 (13.22 – 19.11)	15.46 (13.00 – 18.56)	14.21 (12.02 – 16.73)	14.26 (12.09 – 16.81)
Zambia	8.38 (7.17 – 9.79)	8.35 (7.11 – 9.69)	8.18 (7.01 – 9.47)	7.96 (6.85 – 9.22)	7.95 (6.83 – 9.19)
**Case-detection ratio**					
Cambodia	71% (59% – 86%)	69% (58% – 83%)	68% (57% – 82%)	66% (55% – 79%)	64% (54% – 76%)
Ethiopia	90% (83% – 97%)	90% (83% – 97%)	89% (82% – 96%)	87% (80% – 94%)	87% (80% – 93%)
Kenya	74% (63% – 85%)	73% (63% – 83%)	72% (62% – 82%)	69% (60% – 79%)	68% (59% – 78%)
Lao PDR	28% (22% – 38%)	27% (21% – 35%)	26% (21% – 35%)	24% (19% – 31%)	23% (19% – 30%)
Malawi	71% (59% – 84%)	69% (57% – 82%)	68% (56% – 80%)	64% (53% – 75%)	63% (52% – 74%)
Pakistan	80% (72% – 89%)	79% (71% – 88%)	78% (70% – 86%)	76% (68% – 84%)	75% (67% – 83%)
Philippines	56% (48% – 66%)	55% (47% – 64%)	54% (46% – 63%)	53% (46% – 62%)	52% (45% – 60%)
Tanzania	74% (64% – 83%)	72% (63% – 82%)	71% (62% – 80%)	68% (59% – 77%)	67% (59% – 76%)
Uganda	68% (58% – 79%)	67% (57% – 77%)	66% (56% – 76%)	63% (54% – 73%)	62% (53% – 71%)
Vietnam	67% (55% – 81%)	66% (54% – 78%)	63% (53% – 75%)	59% (50% – 69%)	59% (49% – 69%)
Zambia	84% (74% – 93%)	82% (74% – 92%)	81% (73% – 90%)	79% (71% – 88%)	78% (70% – 87%)
**Incidence rate, per 100,000**					
Cambodia	319 (261 – 380)	331 (272 – 389)	333 (276 – 393)	345 (285 – 406)	353 (296 – 415)
Ethiopia	163 (151 – 176)	164 (152 – 177)	165 (153 – 179)	168 (156 – 183)	170 (157 – 185)
Kenya	337 (290 – 388)	342 (295 – 393)	346 (300 – 398)	359 (311 – 412)	365 (317 – 418)
Lao PDR	511 (370 – 644)	537 (397 – 675)	545 (404 – 682)	595 (454 – 743)	609 (468 – 758)
Malawi	198 (165 – 239)	204 (170 – 245)	208 (175 – 250)	222 (186 – 265)	224 (189 – 268)
Pakistan	270 (243 – 301)	274 (246 – 306)	279 (250 – 309)	287 (258 – 319)	290 (262 – 321)
Philippines	866 (733 – 1,010)	885 (751 – 1,027)	897 (760 – 1,040)	916 (779 – 1,059)	932 (798 – 1,076)
Tanzania	283 (248 – 323)	288 (253 – 330)	293 (259 – 334)	306 (269 – 349)	309 (272 – 352)
Uganda	326 (280 – 381)	332 (285 – 387)	336 (289 – 393)	353 (303 – 410)	359 (308 – 416)
Vietnam	203 (167 – 243)	208 (173 – 248)	215 (179 – 254)	230 (194 – 272)	232 (197 – 275)
Zambia	402 (360 – 450)	408 (365 – 455)	413 (371 – 460)	425 (382 – 472)	430 (386 – 476)

μ: background mortality based on the World Population Perspects, 2016 for Kenya and 2019 for Blantyre (Malawi)

Table 25: Sensitivity analysis: assumptions on untreated TB mortality rates for people living with HIV

Scenario	N1	N2	N3	N4
**Assumptions**				
HIV status	Non-HIV	PLHIV		
Background mortality	μ	μ + HIV-related mortality		
Excess mortality for symptomatic TB	Smear-: 50% / 10 years<br>Smear+: 70% / 10 years	0	Smear-: 50% / 10 years<br>Smear+: 70% / 10 years	5% per month
**Blantyre**				
**All TB regardless of symptomatic or not**				
Time asymptomatic, month	8.79 (5.14 – 13.18)	1.27 (0.27 – 2.91)	1.32 (0.31 – 3.04)	1.15 (0.25 – 2.64)
Time bacteriologically-positive, month	16.27 (11.59 – 21.53)	3.76 (1.85 – 6.16)	3.76 (1.81 – 6.07)	3.26 (1.76 – 5.03)
Case-detection ratio	76% (64% – 88%)	95% (90% – 98%)	94% (88% – 98%)	84% (74% – 93%)
Incidence rate in 2019, per 100,000	158 (132 – 192)	1,718 (1,608 – 1,845)	1,742 (1,618 – 1,879)	1,949 (1,745 – 2,217)
**All symptomatic TB**				
Time bacteriologically-positive, month	7.47 (4.53 – 11.05)	2.49 (1.03 – 4.40)	2.44 (1.00 – 4.25)	2.11 (0.95 – 3.49)
Case-detection ratio	88% (79% – 95%)	97% (93% – 99%)	95% (90% – 98%)	85% (75% – 94%)
Incidence rate in 2019, per 100,000	136 (121 – 155)	1,690 (1,594 – 1,802)	1,712 (1,604 – 1,831)	1,921 (1,721 – 2,177)
**Kenya**				
**All TB regardless of symptomatic or not**				
Time asymptomatic, month	10.35 (8.41 – 12.84)	3.83 (2.42 – 5.50)	3.81 (2.34 – 5.51)	3.22 (1.99 – 4.75)
Time bacteriologically-positive, month	20.90 (18.01 – 24.46)	8.03 (5.92 – 10.36)	7.79 (5.78 – 9.99)	6.49 (4.94 – 8.11)
Case-detection ratio	69% (59% – 81%)	86% (78% – 92%)	84% (77% – 91%)	71% (63% – 80%)
Incidence rate in 2019, per 100,000	263 (221 – 306)	1,987 (1,846 – 2,166)	2,025 (1,873 – 2,205)	2,403 (2,142 – 2,715)
**All symptomatic TB**				
Time bacteriologically-positive, month	10.56 (8.86 – 12.48)	4.20 (2.65 – 6.05)	3.99 (2.52 – 5.56)	3.27 (2.16 – 4.40)
Case-detection ratio	83% (75% – 91%)	92% (87% – 96%)	90% (85% – 95%)	76% (67% – 84%)
Incidence rate in 2019, per 100,000	218 (198 – 240)	1,845 (1,765 – 1,958)	1,882 (1,791 – 2,003)	2,260 (2,025 – 2,542)

μ: background mortality based on the World Population Perspects, 2016 for Kenya and 2019 for Blantyre (Malawi)
HIV-related mortality: AIDS-related deaths / PLHIV for aged 15 and above from the UNAIDS data
5% per month (0.61 per year): an extreme case for gold miner in pre-ART era

E.2 Symptomatic definition

For Blantyre and Kenya with individual-level data, we tested the sensitivity analysis on the criteria defining symptomatic TB.

Table 26: Sensitivity analysis: definition of symptomatic TB

Scenario	S0	S1	S2
Symptomatic	Cough >= 2 weeks	Definition 2	Definition 3
**Blantyre**			
**All TB regardless of symptomatic or not**			
Time asymptomatic, month	4.45 (2.70 – 6.65)	2.96 (1.56 – 4.73)	
Time bacteriologically-positive, month	8.87 (6.42 – 11.67)	8.55 (6.20 – 11.16)	
Case-detection ratio	85% (77% – 93%)	85% (77% – 92%)	
Incidence rate in 2019, per 100,000	382 (346 – 427)	383 (347 – 427)	
**All symptomatic TB**			
Time bacteriologically-positive, month	4.40 (2.50 – 6.39)	5.60 (3.71 – 7.78)	
Case-detection ratio	91% (85% – 96%)	89% (81% – 94%)	
Incidence rate in 2019, per 100,000	357 (334 – 387)	367 (340 – 401)	
**Kenya**			
**All TB regardless of symptomatic or not**			
Time asymptomatic, month	8.80 (7.17 – 10.68)	6.00 (4.71 – 7.53)	1.43 (0.93 – 2.09)
Time bacteriologically-positive, month	17.74 (15.37 – 20.41)	17.32 (15.06 – 20.04)	16.25 (13.96 – 18.91)
Case-detection ratio	69% (59% – 79%)	68% (59% – 78%)	68% (58% – 77%)
Incidence rate in 2019, per 100,000	358 (311 – 414)	363 (317 – 417)	365 (321 – 421)
**All symptomatic TB**			
Time bacteriologically-positive, month	8.91 (7.61 – 10.39)	11.30 (9.80 – 13.09)	14.81 (12.69 – 17.29)
Case-detection ratio	80% (73% – 87%)	75% (68% – 83%)	69% (60% – 78%)
Incidence rate in 2019, per 100,000	308 (284 – 337)	328 (298 – 364)	357 (317 – 408)

Definition 2: cough >= 14 days, hemoptysis, fever, weight loss, night sweats

Definition 3: cough >= 14 days, hemoptysis, fever, breathe shortness, hemoptysis, chest pains, night sweats, weight loss

E.3 Symptom reversion rate sensitivity analyses

In our main analyses, we assume that symptom progression occurs in one direction only. Here, we consider the possibility of reversion from the symptomatic state to asymptomatic state and assess the potential impacts on mis-specified inference.

E.3.1 Theoretical model

We start with Model A with the steady declining condition, and we add a reversion term with a rate of δ. That is,

$$
\begin{aligned}
\frac{\mathrm{d}N_A}{\mathrm{d}t} &= \Lambda - (\theta + \gamma_A)N_A + \delta N_S && = -\phi N_A \\
\frac{\mathrm{d}N_S}{\mathrm{d}t} &= \theta N_A - (\rho + \gamma_S)N_S - \delta N_S && = -\phi N_S \\
N_A + N_S &= N
\end{aligned}
$$

The ratios of prevalent cases by states are

$$N_A : N_S = (\rho + \gamma_S + \delta - \phi) : \theta$$

By assuming a deterministic presentation of case notification, prevalent A, N_A, and prevalent S, N_S, at the survey year, the relation between data and parameters of development rate, θ, and care-seeking rate, ρ are

$$
\begin{aligned}
\rho &= \frac{Notification}{N_S} \\
\theta &= \frac{N_S(\hat{\rho} + \gamma_S + \delta - \phi)}{N_A} \\
&= \frac{Notification}{N_A} + \frac{N_S(\gamma_S + \delta - \phi)}{N_A}
\end{aligned}
$$

However, the point estimates (denoted with hats) of θ and ρ given γ_A, γ_S, ϕ with the model ignoring symptom reversion are

$$
\begin{aligned}
\hat{\rho} &= \frac{Notification}{N_S} \\
\hat{\theta} &= \frac{Notification}{N_A} + \frac{N_S(\gamma_S - \phi)}{N_A}
\end{aligned}
$$

Therefore, the biases of the estimation caused by ignoring symptom reversion can be written as

$$
\begin{aligned}
bias_\rho &= \hat{\rho} - \rho = 0 \\
bias_\theta &= \hat{\theta} - \theta = -\frac{N_S}{N_A}\delta
\end{aligned}
$$

As a result, as reversion rate δ increases, the estimation of symptom development rate $\hat{\theta}$ assum-

ing no reversion decreases linearly (with gradient N_S/N_A). The value of reversion rate has no impact on the care-seeking rate. However, since the reversion is a competing event of care-seeking, the duration of symptomatic will be shorter. Therefore, the estimation of duration ignoring the reversion will overestimate the duration prior to case-seeking.

Healthcare cascade in asymptomatic stage Here, we used $dropout_A$, $dropout_S$, *detected*, and *reversion* to indicate the events of dropout in asymptomatic stage, dropout in symptomatic stage, case notification and symptom reversion, and *rev* to indicated the number of reversion happened before leaving the system. The probabilities of each event by stage are

$$
\begin{aligned}
pr(dropout_A|A) &= \frac{\gamma_A}{\theta + \gamma_A} \\
pr(dropout_A, rev = 0) &= pr(dropout_A|A) \\
pr(dropout_S|S) &= \frac{\gamma_S}{\rho + \gamma_S + \delta} \\
pr(dropout_S, rev = 0) &= (1 - pr(dropout_A|A))pr(dropout_S|S) \\
pr(detected|S) &= \frac{\rho}{\rho + \gamma_S + \delta} \\
pr(detected, rev = 0) &= (1 - pr(dropout_A|A))pr(detected|S) \\
pr(reversion) &= \frac{\delta}{\rho + \gamma_S + \delta}
\end{aligned}
$$

For dropout in asymptomatic stage, the event probabilities by the number of reversions are

$$
\begin{aligned}
pr(dropout_A, rev = 0) &= pr(dropout_A|A) \\
pr(dropout_A, rev = 1) &= pr(dropout_A|A)(1 - pr(dropout_A|A))pr(reversion) \\
pr(dropout_A, rev = 2) &= pr(dropout_A|A)\big((1 - pr(dropout_A|A))pr(reversion)\big)^2 \\
&\cdots \\
pr(dropout_A, rev = n) &= pr(dropout_A|A)\big((1 - pr(dropout_A|A))pr(reversion)\big)^n
\end{aligned}
$$

Then,

$$
\begin{aligned}
pr(dropout_A) &= \sum_{n \geq 0} pr(dropout_A, rev = n) \\
&= pr(dropout_A|A) \sum_{n \geq 0} \big((1 - pr(dropout_A|A))pr(reversion)\big)^n \\
&= pr(dropout_A|A) \frac{1}{1 - (1 - pr(dropout_A|A))pr(reversion)}
\end{aligned}
$$

Let $K = \frac{1}{1-(1-pr(dropout_A|A))pr(reversion)} = \frac{1}{1-\frac{\theta}{\theta+\gamma_A}\frac{\delta}{\rho+\gamma_S+\delta}}$, which increases as δ became larger,

$$
\begin{aligned}
pr(dropout_A) &= \frac{\gamma_A}{\theta + \gamma_A} K \\
&> \frac{\gamma_A}{\theta + \gamma_A}
\end{aligned}
$$

Likewise, the probabilities dropout in symptomatic stage and case-detection are therefore,

$$
\begin{aligned}
pr(dropout_S) &= pr(dropout_S | rev = 0) K \\
&= \frac{\theta}{\theta + \gamma_A} \frac{\gamma_S}{\rho + \gamma_S + \delta} K \\
&= \frac{\theta \gamma_S}{(\theta + \gamma_A)(\rho + \gamma_S + \delta) - \theta\delta} \\
&= \frac{\theta \gamma_S}{(\theta + \gamma_A)(\rho + \gamma_S) + (\theta + \gamma_A)\delta - \theta\delta} \\
&= \frac{\theta \gamma_S}{(\theta + \gamma_A)(\rho + \gamma_S) + \gamma_A \delta} \\
&< \frac{\theta}{\theta + \gamma_A} \frac{\gamma_S}{\rho + \gamma_S} \\
pr(detected) &= pr(detected | rev = 0) K \\
&= \frac{\theta}{\theta + \gamma_A} \frac{\rho}{\rho + \gamma_S + \delta} K \\
&< \frac{\theta}{\theta + \gamma_A} \frac{\rho}{\rho + \gamma_S}
\end{aligned}
$$

As a result, as reversion rate δ increases, the probability of dropout while asymptomatic increases while the probabilities of dropout in symptomatic stage and case notification decrease. The probability of dropout in asymptomatic stage will be under-estimated while the other two events will be over estimated. The probability of dropout in asymptomatic stage with $\delta = 0$ can be identified as the probability of dropout before *ever* developing symptoms when $\delta > 0$.

E.3.2 Simulation study

Study design We prepare three test sets by assuming different rates of symptom reversion: 0, 0.1, and 0.3 per year considering the magnitude of self-cure rate. Given a scenario with a test set and true values of ρ and θ, we set up 500 experiments to address the bias from ignoring the symptom reversion. For each experiment, we generate a 10-years time-series data of case notification and prevalent cases at the fifth year and estimate the maximum a posteriori (MAP) of ρ and θ with Model A. Lastly, We evaluate the mean squared error between true value and the MAP estimators for each scenario. For the true values of parameters, ρ and θ consider the combination of range from 0.5 to 1.5 by 0.5 per year. The other parameters are listed as follows:

Results Figure E.3.2 shows the estimates of symptom development rate ($\hat{\theta}$) and care-seeking rate ($\hat{\rho}$) and Figure E.3.2 shows the estimation error related to the symptom reversion. The existence of symptom reversion did not bias the estimation of care-seeking rate but higher reversion rate in the underlying model causes the symptom development rate being under-estimated. The impact of the reversion on $\hat{\theta}$ are stronger along with lower care-seeking rate in the underlying model. When the symptom reversion

Parameter	Description	Value
ϕ	annual decline rate for incidence	0.05
ν	self-cure rate	0.2
μ	background death rate	0.015
μ_S	excess death rate of untreated symptomatic TB	0.1
γ_A	$\nu + \mu$	0.215
γ_S	$\nu + \mu + \mu_S$	0.315
	Population size	1e7
	Initial prevalence	1%

rate reach 0.3 per year, the bias of symptom development rate estimation can be up to 30% within the considered scenarios.

In terms of overall duration of asymptomatic and symptomatic stages, Figure E.3.2 shows Model A over-estimated the duration in each stage while symptom reversion exists. The errors of duration of asymptomatic stage are higher with higher symptom development rates or lower care-seeking rate; that of symptomatic stage are higher with lower care-seeking rate regardless the magnitude of symptom development rate.

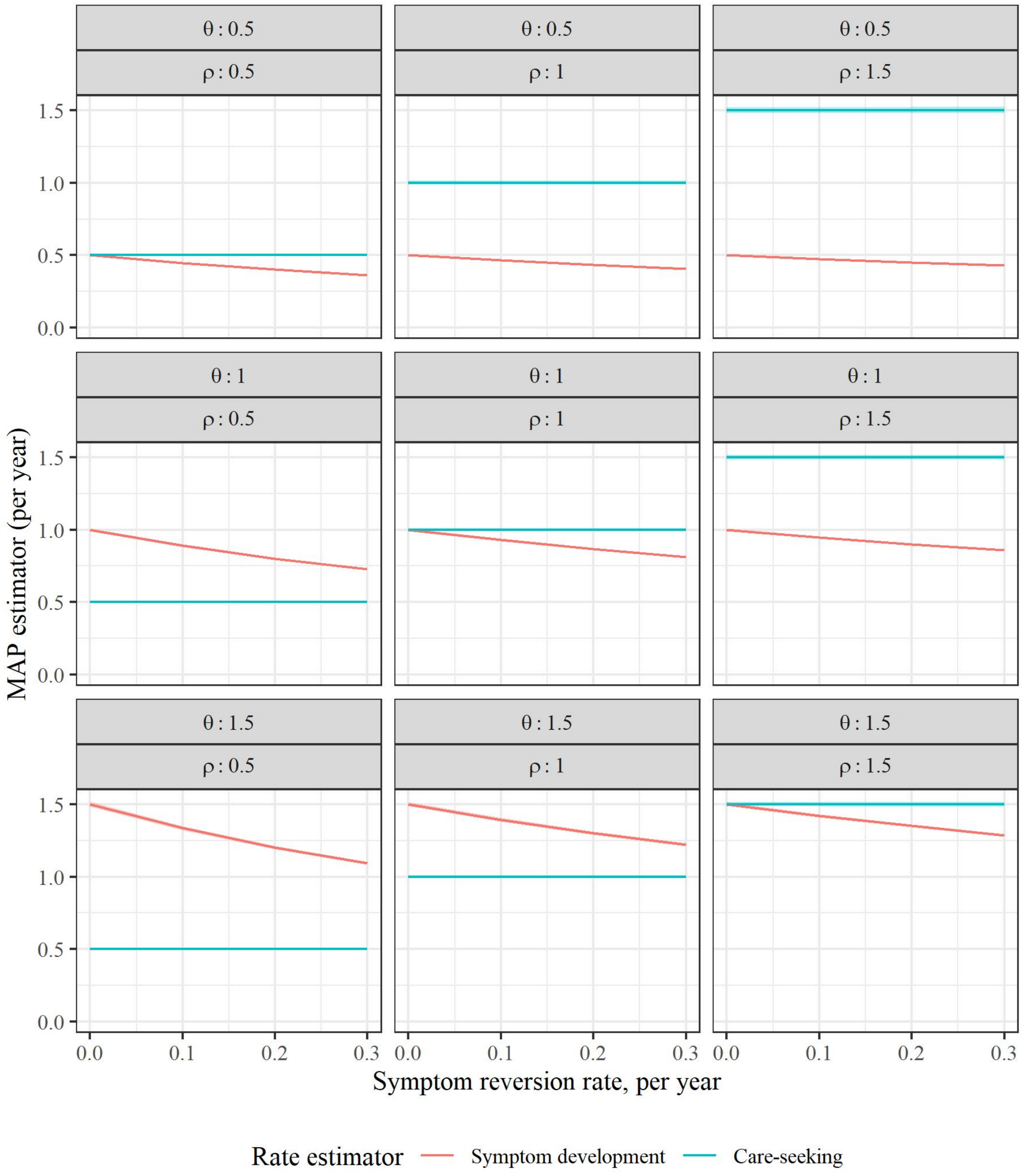

Figure 40: Model estimates given symptom development rate (θ) and care-seeking rate (ρ). The bandwidth shows the 95% credible intervals of estimators

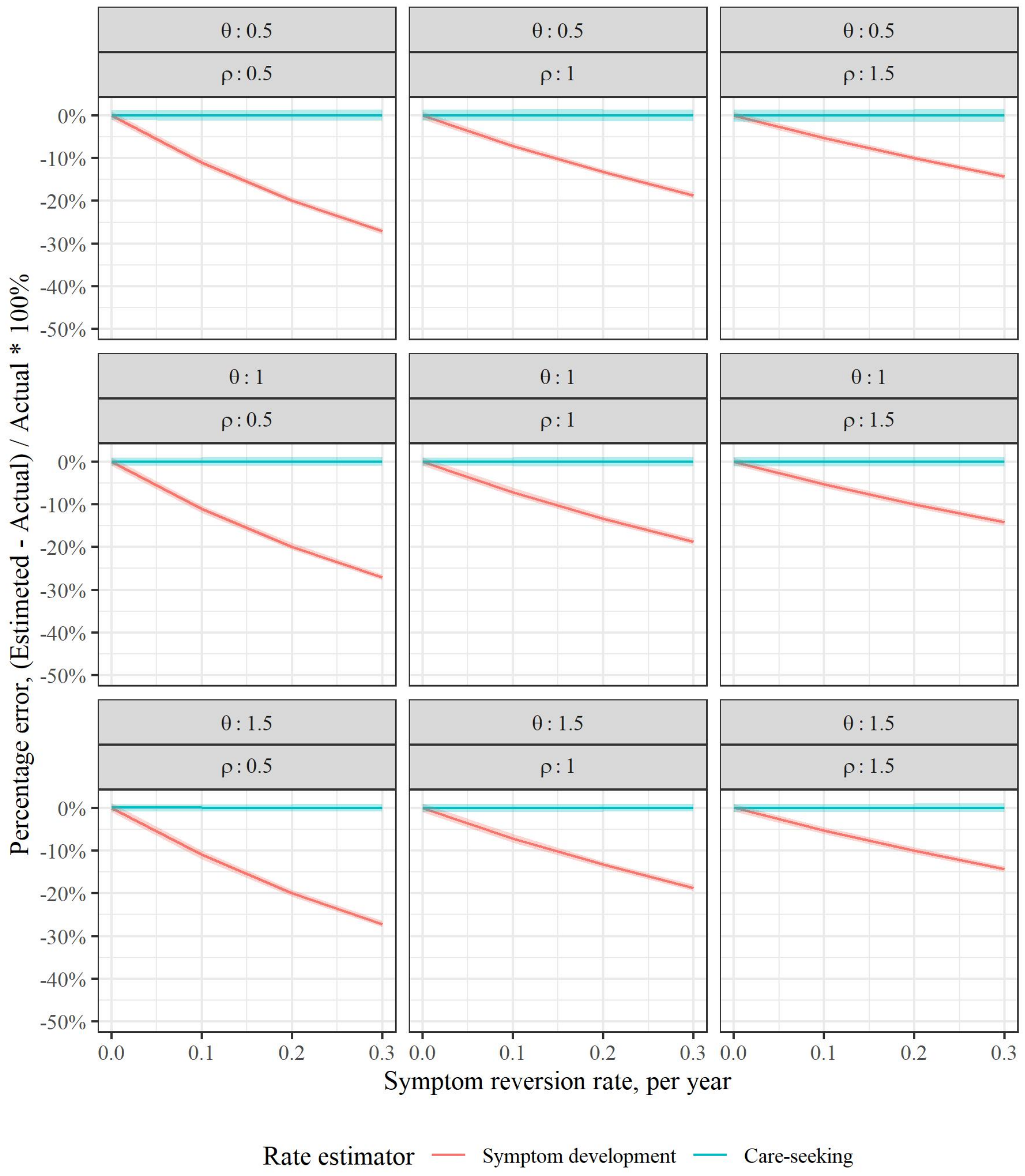

Figure 41: Prediction errors of rates in percentage given symptom development rate (θ) and care-seeking rate (ρ). The bandwidth shows the 95% credible intervals of estimators

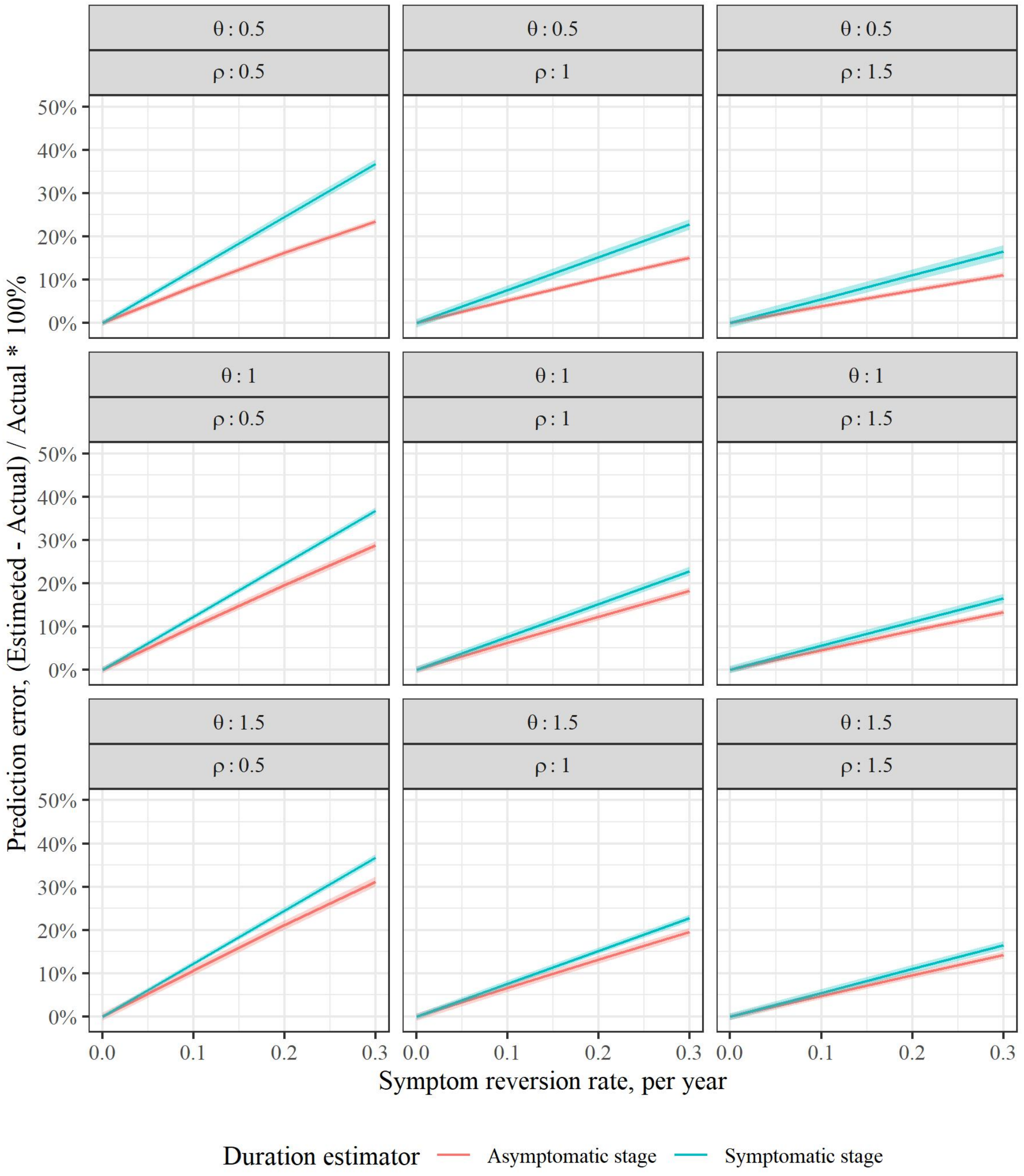

Figure 42: Prediction errors of duration in percentage given symptom development rate (θ) and care-seeking rate (ρ). The bandwidth shows the 95% credible intervals of estimators
